# Characterization of Enterovirus Associated m6A RNA Methylation in Children With Neurological Symptoms: A Prospective Cohort Study

**DOI:** 10.3389/fnins.2021.791544

**Published:** 2021-12-07

**Authors:** Danping Zhu, Yongling Song, Dandan Hu, Suyun Li, Guangming Liu, Peiqing Li, Sida Yang

**Affiliations:** ^1^Department of Pediatric Emergency, Guangzhou Women and Children’s Medical Center, Guangzhou Medical University, Guangzhou, China; ^2^Department of Pediatric Neurology, Guangzhou Women and Children’s Medical Center, Guangzhou Medical University, Guangzhou, China

**Keywords:** enterovirus, neurological symptoms, children, N6-methyladenosine (m6A), m(6)A-RNA immunoprecipitation sequencing

## Abstract

Little is known about the particular changes of N6-methyladenosine (m6A) RNA methylation in enterovirus (EV) infection among children with neurologic symptoms. Here, we determined the characterization of EV associated m6A RNA methylation in this population. A prospective cohort study was conducted from 2018/2 to 2019/12 at the Guangzhou Women and Children’s Medical Center. We included EV infected children with and without neurological symptoms. High-throughput m(6)A-RNA immunoprecipitation sequencing (MeRIP-seq) and RNA-seq analysis were used to evaluate the m6A RNA methylation and transcript expression of cerebrospinal fluid samples. The functional annotation and pathways of differentially methylated m6A genes with synchronously differential expression were analyzed by Gene Ontology (GO) and Kyoto Encyclopedia of Genes and Genomes (KEGG). Seven patients were enrolled in the control group, and 13 cases were in the neurological symptoms (NS) group. A total of 3472 differentially expressed genes and 957 m6A modified genes were identified. A conjoint analysis of MeRIP-seq and RNA-seq data found 1064 genes with significant changes in both the m6A modifications and mRNA levels. The different m6A RNA methylation was increased in the transcriptome’s CDS regions but decreased in both the 3′UTRs and stop codon among the NS group. Functional annotation like the “oxidative phosphorylation” gene pathway, “Parkinson’s disease” and GO terms like “respiratory electron transport chain,” “cellular metabolic process,” and “oxidation-reduction process” was enriched in symptomatic patients. Our study elucidated the changes of RNA m6A methylation patterns and related cellular functions and signaling pathways in EV patients with neurologic symptoms.

## Introduction

Enteroviruses (EV) are common pathogens that causes an array of diseases, especially in neonates. This includes hand-foot and mouth disease (HFMD), aseptic meningitis, encephalitis, acute flaccid paralysis, and acute flaccid myelitis (Gonzalez et al., 2019). It has several genotypes, among which, EV-A71, CV-A6 and CV-A16 have caused large epidemics in Asia since 1997 (Lee et al., 2014; Wang et al., 2017; Huang et al., 2018). Recently, a multicenter prospective study found that the leading cause of viral encephalitis and meningitis in Chinese children was the human EV (Ai et al., 2017). Enteroviruses infection is known to cause thalamus and medulla oblongata damage, thus induces a persisting neurological sequelae or even death (Gonzalez et al., 2019). Therefore, the treatment for EV infections remained mainly supportive apart from the recent anecdotal use of small molecules like ribavirin and DTriP-22 that inhibit EV-A71 by blocking the polymerase (Li et al., 2008). Although our understanding of the molecular mechanisms involved in EV infections has increased dramatically in the past few years, the exact mechanisms of enterovirus infection still remain unknown, especially with regards to the pathogenesis of neurologic damage.

Similar to proteins and DNA, RNA modifications are involved in many aspects of biological functions, like gene expression, protein translation, cell behaviors, and physiological conditions, of which m6A modification of mRNAs is the major form (Du et al., 2019). It has been reported that m6A modification is associated with many human diseases, for example, obesity (Frayling et al., 2007; Ho et al., 2010) and a significant number of cancers (Jin et al., 2012; Reddy et al., 2013; Lin et al., 2016). Also, it plays an important role in brain development as well as a variety of neurological disorders (Du et al., 2019). Some studies have identified that m6A tagged genes are associated with the development of mental disorders, intellectual disability, schizophrenia, and bipolar disorders (Yoon et al., 2017). Recently, the association between RNA methylation and viral infections have gained popularity among researchers especially with the advent of transcriptome sequencing technology. Lichinchi et al. (2016b) found that the host RNA methyltransferase plays a negative post-transcriptional regulatory role in ZIKV virus. Some reports also described the process of m6A modification in HIV-1 RNA and its mechanisms in which it affects viral gene expression (Kennedy E et al., 2016; Lichinchi et al., 2016a). Despite ongoing progress in the field, little is known about the particular changes of m6A RNA methylation in EV infection among children with clinical neurologic symptoms.

In this study, we used RNA-sequencing technology to profile the transcriptome-wide alterations of m6A RNA methylation in cerebrospinal fluid (CSF) samples of simple enterovirus infected children with and without neurologic symptoms, with the aim of understanding the underlying mechanisms of neurologic damage caused by EV infections. This project used the SingleSiteMod program to perform single-base site analysis. The aim was to find the position of the “GGAC” sequence in the m6A peak region, which will reflect the exact position and number of m6A modifications in a peak region. This result has an important reference value for studying m6A modification. We hope this study will contribute to highlighting the pathogenesis of EV infections and will further provide countermeasures to mitigate disease chains of transmission.

## Materials and Methods

### Ethics Approval and Consent to Participate

This study was approved by the Ethics Committee of the Guangzhou Women and Children’s Medical Center (No.2017122501 and 2021019A01). All procedures performed involving human participants were in accordance with the ethical standards of the institutional and/or National Research Committee and in line with the 1964 Helsinki declaration. All patients signed an informed consent form upon admission.

### Study Subjects and Sampling

A descriptive, prospective cohort study was conducted from January 2018 to December 2019 in the Guangzhou Women and Children’s Medical Center. Detailed demographic, clinical characteristics at admission, biochemistry, CSF, and hematologic indicators of the enrolled patients were extracted from the structured electronic medical records system (EMRS). The earliest value of indicators within 24 h after admission was used. Pediatric patients whose CSF samples were collected within 24 h of admission were enrolled. Patients with either positive throat or anal swabs or those with a positive CSF EV-Rt-PCR result were confirmed as EV infection (Zhou et al., 2015). We included all children with EV infection, including those with and without neurological symptoms. Among the patients with neurology involvement (NS group), convulsions, vomiting, headache or altered state of consciousness occurred showing in Table 1. The patients who had fever of unknown origin (FUO) or infection lesions were not clear with high inflammatory indicators had undergone the CSF test and was divided into control group. We excluded those coinfected with other pathogens. Patients with or without neurologic symptoms were matched for age to reduce bias, and then divided into control and NS group.

**TABLE 1:** Demographic and clinical characteristics of enrolled patients.

Groups	All (*n* = 20)	Control group (*n* = 7)	NS group (*n* = 13)	*P*-value
Sex female, n (%)	9 (45)	2 (28.6)	7 (53.8)	0.29
Age, months (IQR)	18 (2, 26)	21 (13, 24)	13 (2, 30)	0.66
Weight, mean ± SD, kg	9.52 ± 3.25	10.01 ± 2.28	9.25 ± 3.73	0.63
Onset, mean ± SD, days	2.80 ± 4.25	1.8 ± 1.39	3.3 ± 5.2	0.45
Fever, n (%)	17 (85.00)	5 (71.43)	12 (92.31)	0.22
Convulsions, n (%)	11 (55)	0	11 (84.62)	0
Vomiting, n (%)	2 (10)	0	2 (15.38)	0.29
Headache, n (%)	1 (5)	0	1 (7.69)	0.46
Fatigue/somnolence, n (%)	1 (5)	0	1 (7.69)	0.46
Acute disorder of consciousness, n (%)	1 (5)	0	1 (7.69)	0.46
Aspartate aminotransferase, mean ± SD, (U/L)	44.68 ± 25.29	32.83 ± 3.54	51.15 ± 29.18*	0.04
CSF microprotein, mean ± SD, (g/L)	0.44 ± 0.36	0.25 ± 0.15	0.54 ± 0.41*	0.04
Lymphocytes, mean ± SD (10^9/L)	3.35 ± 1.77	4.24 ± 2.44	2.88 ± 1.12	0.20
Glucose, mean ± SD (mmol/L)	5.88 ± 0.66	6.07 ± 0.77	5.79 ± 0.62	0.42
Immunoglobulin E, mean ± SD (IU/ML)	72.67 ± 140.43	137.33 ± 229.31	40.33 ± 56.18	0.35
Neuroimaging abnormal findings, n (%)	5/13(38.46)	0	5/8(62.50)	0.07
EEG abnormal findings, n (%)	5/9(55.56)	1/3(33.33)	4/6(66.67)	0.63

* The difference between the EV infected patients with and without neurological symptoms were statistically significant, P<0.05.

Pharyngeal swabs, anal swabs, and CSF samples were collected for detection of EV. Real-time reverse transcription-polymerase chain reaction (RT-PCR) was performed using enterovirus dual fluorescent quantitative RT-PCR kit (Guangdong huayin pharmaceutical technology co. LTD, China). For the clinical routine biochemical testing, we used an automatic analyzer (Automatic Analyzer 7600, Hitachi High-Technologies Corporation). An automatic blood and body fluid analyzer (Sysmex XN550, Japan) was used for the detection of serum and CSF cells.

### RNA Library Construction and Sequencing

For transcriptome profiling, the CSF samples were thawed at 37°C and centrifuged for 30 min at 2000 × *g* at 4°C to clear cells. Then the supernatant was transferred to a new tube and centrifuged at 10,000 × *g* at 4°C for 45min to remove cell fragment. After that, the supernatant was filtered by 0.45 μm filter membrane, and the filtrate was collected. RNA was then extracted from the filtrate and purified by the Zymo RNA clean and concentrator-5 kit (Zymo Research, cat. no R1013). Purified RNA was cut into 200 nt lengths by hyperthermia and added into the precipitation buffer containing the anti-m6A Antibody (Sigma-Aldrich: ABE572), dynabeads Protein A (Invitrogen™, cat. no 10002D) and dynabeads Protein G (Invitrogen™, cat. no 10004D). After magnetic separation and supernatant removal, RNA enzyme inhibitor was added and incubated at 4°C for 1-3 h, and then washed three times by low and high salt precipitation buffer. RNA in the eluate was purified and used for library generation. The obtained products were sequentially removed by ribosomal RNA and synthesized by SMART technique into the first strand cDNA then amplified by PCR and enriched by library fragments, and then DNA purified magnetic bead library fragments were obtained to detect the library by ultrahigh RNA methylation of m6A. Bioptic Qsep100 Analyzer was then used for quality control of the completed libraries.

Sequencing was performed using NovaSeq PE150 in accordance with the manufacturer’s recommendations. HISAT2 software (version 2.1.0) was used to compare the filtered clean reads with the reference genome of the corresponding species of the sample to obtain unique mapped reads for further analysis. A R/Bioconductor package “exomePeak” was used for peak calling. *P* < 0.05 was considered to be differentially peaks distribution. We used HOMER software (version 4.10.4) to perform motif analysis on the Peaks^1^. SingleSiteMod program was used to determine the position and number of m6A modification in the peak region. The raw sequence reads were deposited into the Genome Sequence Archive (GSA) database (File number: OMIX716).

### Conjoint Analysis of MeRIP-seq and RNA-seq Data

To identify the genes with significant changes in both the m6A modification and mRNA levels, conjoint analysis of MeRIP-seq and RNA-seq (MeRIP-seq input library data) data was performed.

The expression level of each gene was calculated by determining the length of the fragments and the read counts mapped to those fragments and were further normalized by a variation of the FPKM method. In our study, the differentially expressed genes were screened and sorted according to log 2FC (log 2FC > 0, up-regulated genes; log 2FC < 0, down-regulated genes), P value, FDR of RNA-sequence difference. The “exomePeak” was used for differential RNA methylation analysis. *P* < 0.05 was considered to be differentially peaks distribution.

### Gene Ontology Annotations and Kyoto Encyclopedia of Genes and Genomes Enrichment

The Gene Ontology (GO) was used to identify the functional annotation and the classification of molecular functions, together with the biological processes and cellular component aspects of the identified m6A methylated RNAs and the differentially m6A methylated RNAs. The Kyoto Encyclopedia of Genes and Genomes (KEGG) database was applied to identify pathways of differentially expressed genes (DEGs) in this study. Meanwhile, Fisher’s exact test was used to calculate the P-value according to the annotations, and the rich factor was calculated based on the numbers of symbols in the list.

### Public Databases and Analysis

We downloaded 31 human tissues from Genotype-Tissue Expression (GTEX), covering the expression levels of 60498 genes (FPKM). According to the method in a previous report (Gerstberger et al., 2014), the tissue specificity score (S) of each gene was calculated and its value ranged from 0 to 4.95. The higher the value, the higher the tissue specificity. *S* ≥ 1 was considered tissue specific, while *S* < 0.3 was considered to be widely expressed. When 0.3 ≤ S < 1, the tissue-specific was at the intermediate level. In this study, the tissue specific genes with *S* ≥ 1 were screened for display, and the heat map was drawn with FPKM value.

### Statistical Analysis

Statistical analysis of the demographic and the clinical data was performed using SPSS version 22.0 (IBM Corp., Armonk, NY, United States). When the data were normally distributed, we expressed it as a mean ± SD; if not, we expressed it as the median and the interquartile range (IQR). P-value of < 0.05 was considered statistically significant.

For analysis of the sequenced data difference, the absolute value of diff. log 2FC ≥ 1, and a *P* < 0.05 were set as the threshold for DEGs. For the analysis of m6A data difference, the input RNA and m6A RNA reads were aligned to genome reference sequences using HISAT2 software. ExomePeak was used for peak calling and differential RNA methylation analysis. HOMER (version 4.10.4) was used for motif search. FDR ≤ 0.05, *P* < 0.05, and diff.log 2FC > 0 was considered as hypermethylation, while diff. log2FC < 0 was considered as demethylation. The absolute value of diff. log 2FC ≥ 1, *P* < 0.05 were set as the threshold for differential m6A methylated RNAs. The global distribution of hypermethylated and hypomethylated m6A peaks was displayed by Circos plot using CIRCOS.

## Results

### Demographic and Clinical Characteristics of Enrolled Patients

**FIGURE 1:**
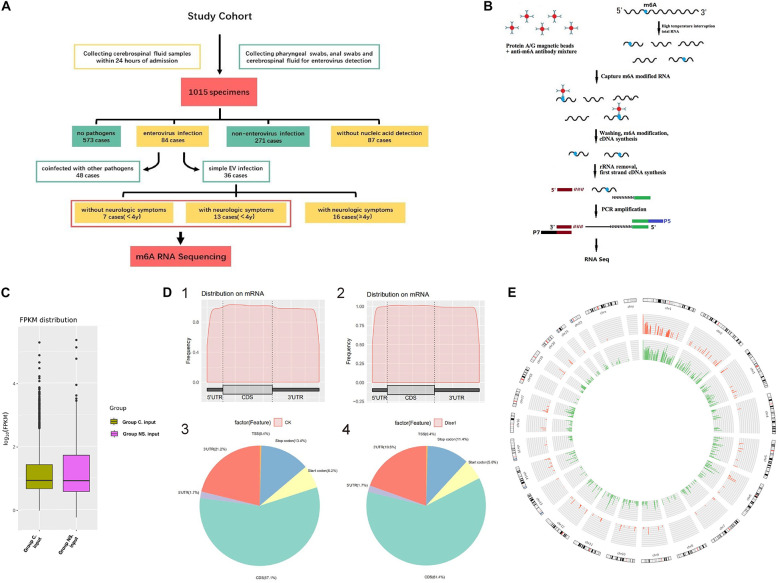
**(A)** Flow chart of patient enrollment. **(B)** The flowchart of meRIP- Sequencing. **(C)** FPKM distribution in control group and NS group. Fragments Per Kilobase of transcript sequence per Millions base pairs. **(D)** m6A RNA Methylation Peaks Distribution of Host Cell RNA Transcripts. 1. m6A peaks distribution on mRNA of EV infected patients without neurological symptoms (control group). 2. m6A peaks distribution on mRNA of EV infected patients with neurological symptoms (NS group). 3. Pie chart of m6A peaks distribution in control group. 4. Pie chart of m6A peaks distribution in NS group. **(E)** Circos plot showing the distribution of hypermethylated and hypomethylated m6A peaks in the human transcriptome of neurological symptoms group compared with control group. Red mark represents the distribution of m6A peaks in NS group. Green mark represents the distribution of m6A peaks in control group. The line in red or green represents an enrichment fold of m6A methylation peaks in different chromosomes with fold change at Y-axis. Reference genome: GRCh38/hg38.

A total of 1015 CSF specimens were collected, in which, 573 cases had no detectable pathogens, 271 cases were non-EV infections, 87 cases had no nucleic acid detection, and 84 cases with EV infection. Among the 84 cases with confirmed EV, 48 cases were coinfected with other pathogens and 36 cases were simple EV infection. In those with simple EV infection, patients without neurologic symptoms (7 cases) were all under 4 years old, so we choose the remaining 13 cases with neurologic symptoms under 4 years old for comparison in order to reduce bias (Figure 1A).

Demographic and clinical information of the enrolled patients was shown in Table 1 and Supplementary Table 1. There were no statistically significant differences in any of the demographic characteristics between the EV infected patients with neurological symptoms (NS group) and those without (control group). The main neurological symptoms of EV infected patients were convulsions, vomiting, headache, dizziness, fatigue/somnolence, and acute disorder of consciousness (ADOC), of which convulsions was the most common. Serum levels of aspartate aminotransferase (AST) were significantly higher in patients with neurological symptoms (*P* = 0.044), and those with a higher concentration of microprotein in CSF samples (*P* = 0.035). The lymphocytes count, immunoglobulin E levels, and glucose concentration were lower in the NS group, but with no statistical significance. In the NS group, eight patients underwent neuroimaging, five of whom had abnormal findings, one had leptomeningeal enhancement and subdural effusion, two patients had abnormal signals located in posterior horn of ventricle or cerebral hemispheres and midbrain, respectively, one had swollen cerebral cortex and basal ganglia, and the last one had enlarged sulci. But generally, most patients had a favorable prognosis and a recovery rate of 69.2%.

Discharge summaries found that, in the control group, 3 cases had hand-foot-and-mouth disease, and 4 cases with herpangina. In the NS group, 4 cases had hand-foot-and-mouth disease or herpangina with febrile convulsions, 5 cases had enterovirus encephalitis, 2 cases had epilepsy with enterovirus infection, one case had enterovirus infection co-infected with bacteria which caused purulent meningitis, and a case with enterovirus infection accompany with Kawasaki disease. All cases in the control group recovered fully, while in the NS group, only 9 cases had a full recovery, 3 cases had clinical improvement and 1 case of death.

### General Features of m6A RNA Methylation

The flowchart of meRIP-sequencing was shown in Figure 1B. The expected number of Fragments Per Kilobase of transcript sequence per Millions base pairs sequenced (FPKM) showed no difference in total gene expression between the two groups (Figure 1C). A total of 957 m6A methylated genes were obtained from the two groups. The distribution of m6A RNA methylation in EV infected children was illustrated in Figure 1D. The m6A peaks of all enrolled patients mainly occurred in the four non-overlapping transcript segments, including coding sequence (CDS), 3′ untranslated regions (3′UTR), stop codon and start codon. Metagene analysis showed that NS patients had an increased level of m6A in the CDS regions of the transcriptome but correspondingly decreased in the 3′UTRs and the stop codon. At the same time, we compared the enrichment folds of m6A methylation peaks in different chromosomes between the NS group and the control group (Figure 1E). No difference in the methylation enrichment on Y chromosome between control group and NS group was observed, while the m6A methylation distribution on other chromosomes was more common in control group than that of the NS group (Figure 1E). The sequence logo of m6A motif was shown in Supplementary Figure 1.

### Conjoint Analysis of MeRIP-seq and RNA-seq Data

We also further investigated changes in the expression pattern of the m6A methylated genes in EV infected patients with neurological symptoms and the control group. A total of 3472 DEGs were identified. The top 20 DEGs according to the absolute log2FC were summarized in Table 2 and the all DEGs were presented in Supplementary Table 2. The DEGs with m6A methylation was indicated in Table 3. Differentially methylated m6A peaks with synchronously differential expression were summarized in Table 4 and Supplementary Figure 3. The m6A methylated genes with synchronously differential expression were identified between the two groups, including PLEKHA4 (Supplementary Figure 2).

**TABLE 2:** Differentially expressed input RNA genes (top 20).

Gene name	Gene ID	Regulation	Gene biotype	Log2FC^#^	FDR	*P*-value
MT-RNR2	ENSG00000210082	Up	Mt-rRNA	1.793355	0	0
MT-ATP6	ENSG00000198899	Down	Protein coding	−4.5763	0	0
RPS23	ENSG00000186468	Down	Protein coding	−5.76759	0	0
RPS15	ENSG00000115268	Down	Protein coding	−9.8552	0	0
MT-CO2	ENSG00000198712	Down	Protein coding	−9.67509	0	0
EMP3	ENSG00000142227	Down	Protein coding	−6.56238	0	0
HSP90AB1	ENSG00000096384	Down	Protein coding	−4.73739	0	0
HSP90AA1	ENSG00000080824	Down	Protein coding	−3.40525	0	0
MAP4	ENSG00000047849	Down	Protein coding	−10.943	0	0
CASC3	ENSG00000108349	Down	Protein coding	−4.70761	0	0
ENO1	ENSG00000074800	Down	Protein coding	−10.1362	0	0
BCL2L1	ENSG00000171552	Down	Protein coding	−10.8885	0	0
PTK2	ENSG00000169398	Down	Protein coding	−6.65357	0	0
MT-ND2	ENSG00000198763	Up	Protein coding	4.865544	0	0
EIF3B	ENSG00000106263	Down	Protein coding	−5.37944	1.41E-299	1.84E-301
STIP1	ENSG00000168439	Down	Protein coding	−6.3886	1.33E-299	1.85E-301
AMOTL1	ENSG00000166025	Down	Protein coding	−9.79476	5.09E-275	7.53E-277
FILIP1L	ENSG00000168386	Down	Protein coding	−13.0964	2.56E-257	4.01E-259
JUNB	ENSG00000171223	Down	Protein coding	−13.0641	3.23E-253	5.34E-255
RAB10	ENSG00000084733	Down	Protein coding	−11.6809	9.90E-251	1.72E-252

*^#^The log2FC is the log2 conversion value of the difference fold change.*

**TABLE 3:** Top 20 of input differentially expressed Genes with m6A methylation in NS group.

Gene name	Locus	Regulation	Gene biotype	Fold enrichment	log2FC	*P*-value
MT-CO2	MT: 7644–7972	Down	Protein coding	55	−9.675089577	0
MT-ND6	MT: 14148–14382	Up	Protein coding	94	4.104219397	2.15E-51
SET	Chr9: 128693653–128693950	Up	Protein coding	82.7	2.104219397	0.014500156
ERICH3	Chr1: 74572124-74572365	Up	Protein coding	61.1	2.939358495	3.68E-199
SFXN3	Chr10: 101040198–101040378	Up	Protein coding	44.2	3.943754725	8.35E-06
PTMA	Chr2: 231711309–231711549	Up	Protein coding	314	4.006039003	1.82E-47
LRRC75A-AS1	Chr17: 16441669–16441849	Up	Processed transcript	39.4	3.810488194	2.68E-09
MT-ATP6	MT: 8526–8793	Down	Protein coding	178	−4.57630233	0
MT-RNR1	MT: 707–946	Up	Mt rRNA	42.5	1.958861618	3.59E-207
PLEKHA4	Chr19: 48868452–48868632	Up	Protein coding	19.3	6.850645321	1.44E-37
PRRC2C	Chr1: 171532482–171532872	Down	Protein coding	1080	−3.366100537	6.08E-05
DDX21	Chr10: 68959968–68960208	Up	Protein coding	142	6.141694103	9.22E-92
MALAT1	Chr11: 65499083–65499293	Down	LincRNA	86	−1.954330752	3.51E-247
SAMHD1	Chr20: 36890438–36890589	Up	Protein coding	6.28	2.036200974	3.75E-11
MT-ND4	MT: 10998–11269	Down	Protein coding	92.9	−2.921315695	8.59E-07
LMNA	Chr1: 156139788–156139969	Up	Protein coding	117	5.726656604	1.22E-18
MT-ND1	MT: 3783–4172	Up	Protein coding	117	1.582116921	1.40E-09
HSP90AB1	Chr6: 44252017–44252226	Down	Protein coding	57.8	−4.737387834	0
MT-RNR2	MT: 2539–3229	Up	Mt rRNA	554	1.793354933	0
RPL18	Chr19: 48617356–48617536	Up	Protein coding	45	7.998398941	2.14E-74

*Fold Enrichment: Fold Enrichment for m6A methylation; Log2FC: Fold change for gene expression.*

**TABLE 4:** Differentially methylated m6A Peaks with synchronously differential expression in NS group.

Gene name	Locus	Regulation	Gene biotype	Fold enrichment	Log2FC^#^	*P*-value
SET*	Chr9:128693801–128693950	Up	Protein coding	82.7	2.104219397	0.0145002
PTMA	Chr2:231711309–231711549	Down	Protein coding	314	4.006039003	1.82E-47
MT-ND6*	MT: 14148–14382	Up	Protein coding	94	4.104219397	2.15E-51
MT-CO2	MT: 7644–7972	Up	Protein coding	55	−9.675089577	0
MT-ND4	MT: 10998–11269	Up	Protein coding	92.9	−2.921315695	8.59E-07
MT-ND1*	MT: 3783–4172	Up	Protein coding	117	1.582116921	1.40E-09
MT-ATP6	MT: 8526-8793	Up	Protein coding	178	−4.57630233	0
MT-RNR2	MT: 2539–3229	Down	Mt rRNA	554	1.793354933	0
MT-RNR1	MT: 707–946	Down	Mt rRNA	42.5	1.958861618	3.59E-207

*Methylated m6A and mRNA were up-regulated at the same time. #The log2FC is the log2 conversion value of the difference foldchange.

Additionally, 31 human tissues, covering the expression levels of 60498 genes were downloaded from the GTEX database among which, the tissue specificity scores of m6A abundant genes in the NS group were shown in Supplementary Figure 3. The heatmap exhibits that PLEKHA4 was a tissue specific gene in nerve and MAP2 was a tissue specific gene in brain (Supplementary Figure 3).

### Gene Ontology Annotation and Kyoto Encyclopedia of Genes and Genomes Pathway Analysis

**FIGURE 2:**
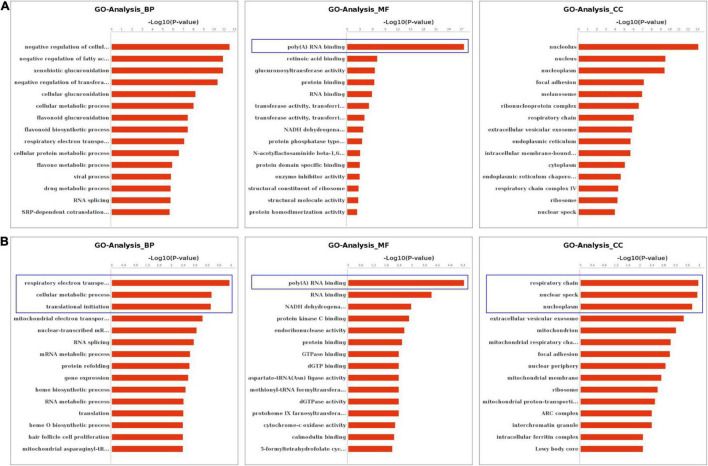
GO Enrichment Analysis of m6A RNA Methylation Genes. **(A)** The top 15 significant enrichment GO terms of genes with m6A modification in control group. **(B)** The top 15 significant enrichment GO terms of genes with m6A modification in neurological symptoms group. NS: neurological symptoms. BP: biological process. MF: molecular function. CC: cellular component.

**FIGURE 3:**
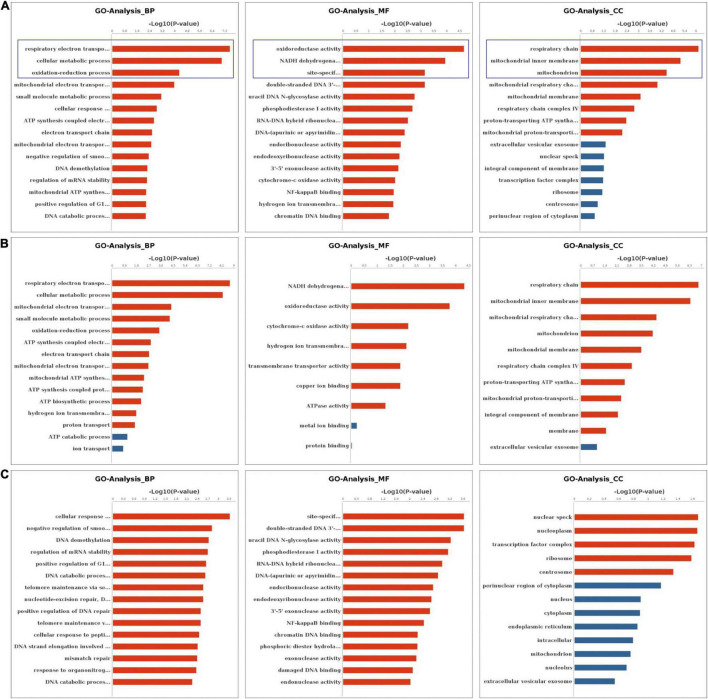
GO Enrichment Analysis of genes with significant changes in both m6A modification and mRNA levels **(A)** The top 15 significant enrichment GO terms of genes with significant changes in both the m6A modifications and mRNA levels in NS group (NS vs. control group). **(B)** The top 15 significant enrichment GO terms of differently m6A methylated genes with significant upregulation of mRNA levels in NS group (NS vs. control group). **(C)** The top 15 significant enrichment GO terms of differently m6A methylated genes with significant downregulation of mRNA levels in NS group (NS vs. control group). NS: neurological symptoms, Red: significant difference, blue: no significant difference. BP: biological process. MF: molecular function. CC: cellular component.

We performed the GO and KEGG pathway analysis to identify the functions of these genes with significant changes in m6A modifications using 15 significant enrichment GO terms in biological process (BP), molecular function (MF) and cellular components (CC) as shown in Figure 2. The GO molecular functional enrichment results showed that the genes with m6A modifications were mainly related to poly(A) RNA binding in all EV infected children especially in those with neurological symptoms (Figures 2A,B). We also annotated the m6A into biological process, and found that, the NS group were mainly involved in the respiratory electron transposition, cellular metabolic process and translational initiation (Figure 2B). Lastly, the cellular component analysis found that, the genes were enriched in the respiratory chain, nuclear speck, and nucleoplasm in NS group, while in the control group, the GO functional classification analysis exhibited distinct results, as shown in Figures 2A,B. Additionally, we performed GO annotation analysis for the genes with significant changes in both the m6A modification and the mRNA levels (Figure 3). Compare with control group (Figure 3A), the GO terms result showed that those genes of NS group were most related to respiratory electron transposition, cellular metabolic and oxidation-reduction in biological process. In molecular function, those genes were mainly involved in oxidoreductase activity, NADH dehydrogenation and site-specificity. We also annotated those genes into cellular component, the results showed that they were enriched in respiratory chain, mitochondrial membrane and mitochondrion (Figure 3A). Furthermore, according to the expression level on mRNA, the differently expressed m6A methylated genes were divided into up-regulation and down-regulation genes, GO annotation of those genes were also performed, respectively, as presented in Figures 3B,C. The result of up-regulation genes was similar with that of genes with significant changes in both m6A modification and mRNA levels (Figures 3A,B), while the down-regulation genes showed distinct results (Figure 3C).

**FIGURE 4:**
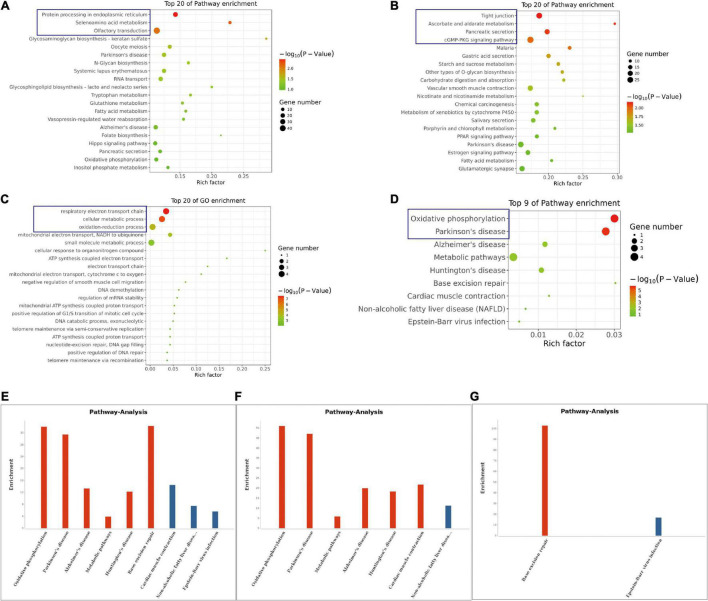
KEGG Pathway Analysis of m6A RNA Methylation Genes. **(A)** The top 20 enriched pathway of genes with m6A modification in control group. **(B)** The top 20 enriched pathway of genes with m6A modification in NS group. **(C)** The top 20 enriched GO terms of the differentially methylated genes in NS group compared with those in control group. **(D)** The top 9 enriched pathways of the differentially methylated genes NS group compared with those in control group. **(E)** The enriched pathways of all genes with significant changes in both the m6A modifications and mRNA levels in NS group (NS vs. Control group). **(F)** The enriched pathways of differently m6A methylated genes with significant upregulation of mRNA levels in NS group (NS vs. Control group). **(G)** The enriched pathways of differently m6A methylated genes with significant downregulation of mRNA levels in NS group (NS vs. Control group).

The KEGG pathway analysis also revealed various top 20 pathway enrichments as shown in Figure 4. In the control group, the protein processing in the endoplasmic reticulum, seienoamino acid metabolism, and the olfactory transduction pathway were significantly enriched (Figure 4A), while in the NS group, the enriched pathways were tight junction, ascorbate and aldarate metabolism, pancreatic secretion, and cGMP-PKG signaling pathways (Figure 4B). Furthermore, GO terms like respiratory electron transport chain, cellular metabolic process and oxidation-reduction process were all enriched as revealed by the analysis of the differentially methylated genes in NS group (Figure 4C). In addition, the KEGG pathway analysis based on the differentially methylated genes between the two groups unveiled that oxidative phosphorylation and Parkinson’s disease pathway were also enriched (Figure 4D). Additionally, the enriched pathways of all genes with significant changes in both the m6A modifications and mRNA levels in NS group were shown in Figures 4E–G.

## Discussion

Globally, EV infection has caused several notable outbreaks in pediatric population due its virulence and mostly in Asian countries. Despite the vast majority of EV infections are mild and self-limiting, some children will develop central nervous complications with serious sequelae and even death. Vaccines developed in China confers protection (Mao et al., 2016), but it is not effective in symptomatic patients. The aim of our study was to explore the underlying mechanism of EV related neurological damage by using RNA-sequencing technology to profile the transcriptome alterations of m6A RNA methylation in CSF samples of simple EV infected children with and without neurologic symptoms. We believe this study will contribute to highlighting the pathogenesis of EV infections and will further provide countermeasures to mitigate disease chains of transmission.

In our cohort, the most prevalent neurological symptoms were convulsions, vomiting, headaches, somnolence, and acute disorder of consciousness (ADOC). We previously found that somnolence in EV patients was correlated with high mortality (Yang et al., 2018). Similarly, previous studies have also shown that symptoms like convulsion, dyspnea, cyanosis, and vomiting were also associated with increased high risk of death from severe HFMD (Long et al., 2016). CVA6-associated severe cases were characterized by high fever with intermittent twitching, while EV71-associated severe cases were characterized by poor mental condition, loss of pupillary reflex, and vomiting (Li et al., 2016; Yang et al., 2017, 2018). However, all our enrolled patients in our study had a favorable prognosis, possibly due to our small sample size. It has been reported that leukocytosis and increased CSF protein is more common in encephalitis than in febrile convulsion cases of HFMD (Xu et al., 2019). In our study, we found a similar pattern of elevated microprotein levels in EV infected patients with neurological symptoms. Contrary to a previously published study on EV-A71 infection associated pulmonary edema (Crabol et al., 2017), there was a low glucose levels and high AST level in the NS group. There is still no consensus on the risk factors associated with severe Infection (Ooi et al., 2009; Crabol et al., 2017; Qin et al., 2019; Xu et al., 2019), hence further well conducted, large sample size studies are needed.

Previous studies have also described the changing patterns of m6A deposition in response to heat shock and Flaviviridae infections, like dengue virus (DENV), Zika virus (ZIKV), West Nile virus (WNV), and hepatitis C virus (HCV) (Zhou et al., 2015; Lichinchi et al., 2016b; Gokhale et al., 2020). Our data indicated that there was a differentially expressed pattern of m6A RNA methylation in EV infected children with neurologic symptoms. We observed changes in the distribution of m6A, with increased peaks in the CDS regions but with a decreased trend in the 3′UTRs and stop codon regions of the children with neurological symptoms. This indicates that EV infection might affect gene translation, gene splicing and mRNA stability by changing the deposition of m6A, leading to the development of severe neurological symptoms and sequalae. Nine m6A methylated genes with synchronously differential expression were identified by conjoint analysis of MeRIP-seq and RNA-seq data. Among which, PLEKHA4, a tissue-specific gene in the nerve, was upregulated and hypermethylated in patients with neurological symptoms. It’s known that PLEKHA4 is involved in autism spectrum disorder (Hashimoto et al., 2016). Previous study demonstrated that PLEKHA4 was a signaling strength modulator in Wnt signaling pathway, which controls key cell fate decisions in the development of multicellular eukaryotes (Shami Shah et al., 2019). One study about Melanoma found that, PLEKHA4 was required for melanoma proliferation and survival, PLEKHA4 knockdown could attenuate tumor growth and enhance the effect of clinically used inhibitor encorafenib (Shami Shah et al., 2021). We speculated that PLEKHA4 and related Wnt signaling may also involve with EV-induced neuron damage. Whether the targeting therapy of PLEKHA4 may help reduce the neuron damage in EV infection needs further study. We infer that those other m6A methylated genes with synchronously differential expression might also participate in EV-induced neuron damage.

We carried out the GO and KEEG pathways analysis of m6A methylated RNAs which showed that, the GO terms including respiratory electron transport chain, cellular metabolic process, and oxidation-reduction process pathways were all enriched in the EV infected patients with neuropsychiatric symptoms, citing a possible correlation with the underlying mechanism of central nervous system damage in EV infections. Recently, an experiment of SH-SY5Y cells with EV71 infection also found a dysregulated transcriptomes profile, which revealed that the enriched GO term “Nervous system development” and enriched pathway “CCKR signaling map” might be important contributors to EV71-induced neuropathological mechanism (Hu et al., 2020). When we compared the difference in signaling pathways of m6A peaks between the children with and without neurological symptoms, we found that, the oxidative phosphorylation and Parkinson’s disease pathway were both enriched, a finding that is consistent with previous study that concluded the association between acute parkinsonism and Coxsackie virus (Jang et al., 2009). Also, another study found the presence of virus-like particles and enterovirus antigen in the brainstem neurons in patients with Parkinson’s disease (Dourmashkin et al., 2018). But the association of EV with aged-onset idiopathic Parkinson’s disease has never been established. One recent study shows that the acute course of EV71-infected patients with neurological symptoms, such as parkinsonian-like “limb tremor” did not persist as a neurological sequela (Yang et al., 2017). Underlying mechanisms need to be elucidated in further studies.

Limitations of our study include the lack of distinct identification of EV serotype among the 100 serotypes of human EV (Khetsuriani et al., 2006). Secondly, this study involves a small number of patients from the same hospital and the results may lack general applicability and extrapolation.

In summary, we described the m6A RNA methylation transcriptomic patterns of EV by high-throughput RNA-sequencing analysis in EV infected children under four years old. We found a variable m6A distribution, pathways, and expression in children with neurological symptoms, which were mostly associated with respiratory chains and cell metabolism. These specific genes with aberrant expression might play an important role in the process and the pathogenesis of CNS lesions caused by EV. Further studies are required to explore the more succinct underlying mechanisms of EV infection.

## Data Availability Statement

The data reported in this paper have been deposited in the OMIX, China National Center for Bioinformation / Beijing Institute of Genomics, Chinese Academy of Sciences (https://ngdc.cncb.ac.cn/omix: accession no.OMIX716).

## Ethics Statement

The studies involving human participants were reviewed and approved by Ethics Committee of the Guangzhou Women and Children’s Medical Center. Written informed consent to participate in this study was provided by the participants’ legal guardian/next of kin.

## Author Contributions

DZ, YS, and DH performed and analyzed the experiments and wrote the manuscript. SL and GL carried out the data collection and data analysis. PL and SY conceived and coordinated the study and designed and revised the manuscript. All authors reviewed the results and approved the final version of the manuscript.

## Conflict of Interest

The authors declare that the research was conducted in the absence of any commercial or financial relationships that could be construed as a potential conflict of interest.

## Publisher’s Note

All claims expressed in this article are solely those of the authors and do not necessarily represent those of their affiliated organizations, or those of the publisher, the editors and the reviewers. Any product that may be evaluated in this article, or claim that may be made by its manufacturer, is not guaranteed or endorsed by the publisher.

## Footnotes

1
http://homer.ucsd.edu/homer/ngs/peakMotifs.html
