# Supplementary material for: Characterization of Enterovirus Associated m6A RNA Methylation in Children With Neurological Symptoms: A Prospective Cohort Study
Source: Front Neurosci. 2021 Dec 7;15:791544. doi: 10.3389/fnins.2021.791544 (PMC8689127; doi:10.3389/fnins.2021.791544)
Supplement: Supplementary file 1 [file Data_Sheet_1.docx]

**Supplementary Figure legends**

**
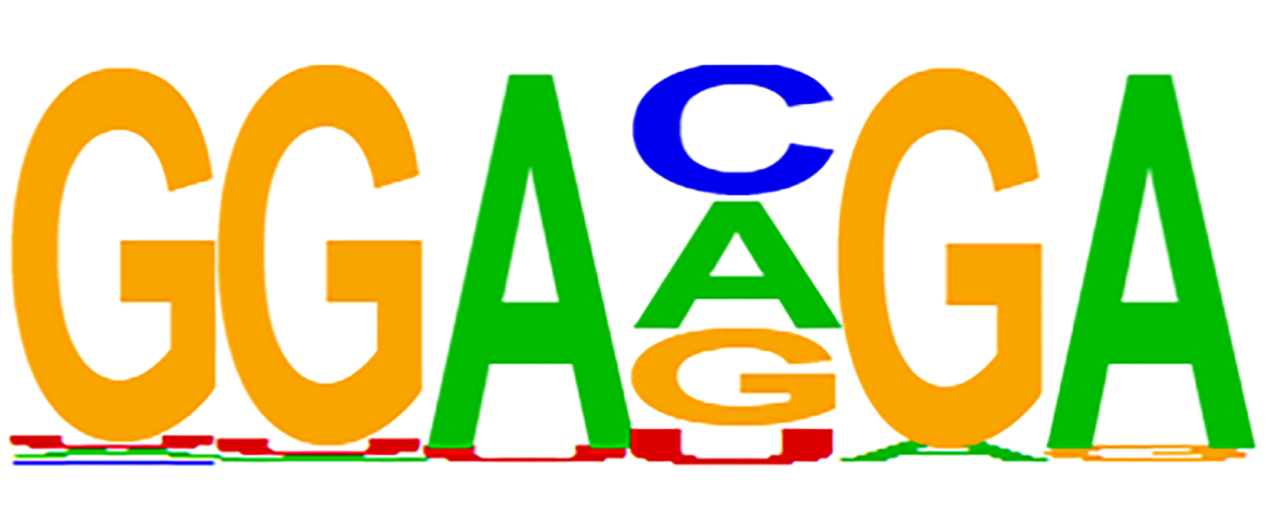
**

**Supplementary Figure 1. Motif analysis of m6A modification in NS group by HOMER.** NS: neurological symptoms.


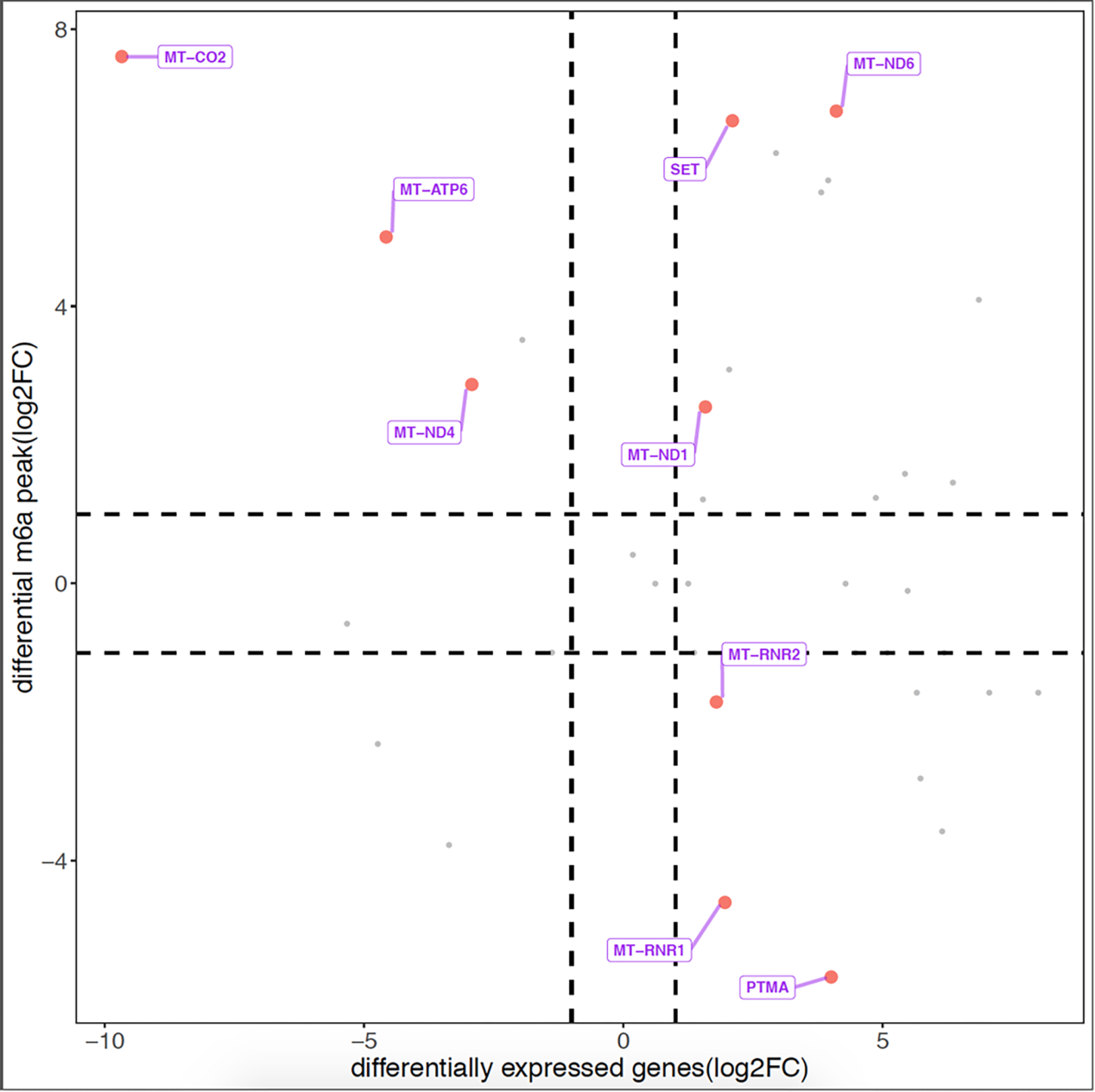


**Supplementary Figure 2. The X-axis represents the log2FC value of gene expression, and the Y-axis represents the log2FC value of m6A methylation.** The top10 genes with the largest variations of m6A were marked in the figure. The red dots represent significant differences (the data of RNA-seq and RNA modification meet |log2FC| ≥ 1, P < 0.05). Gray dots represents points that did not meet the red condition (|log2FC| < 1, or P ≥ 0.05). The mRNA and the m6A-modified genes are up-regulated or down-regulated in the upper right and lower left. The lower right and upper left represent different trends in mRNAtranscription level and m6A modification level.


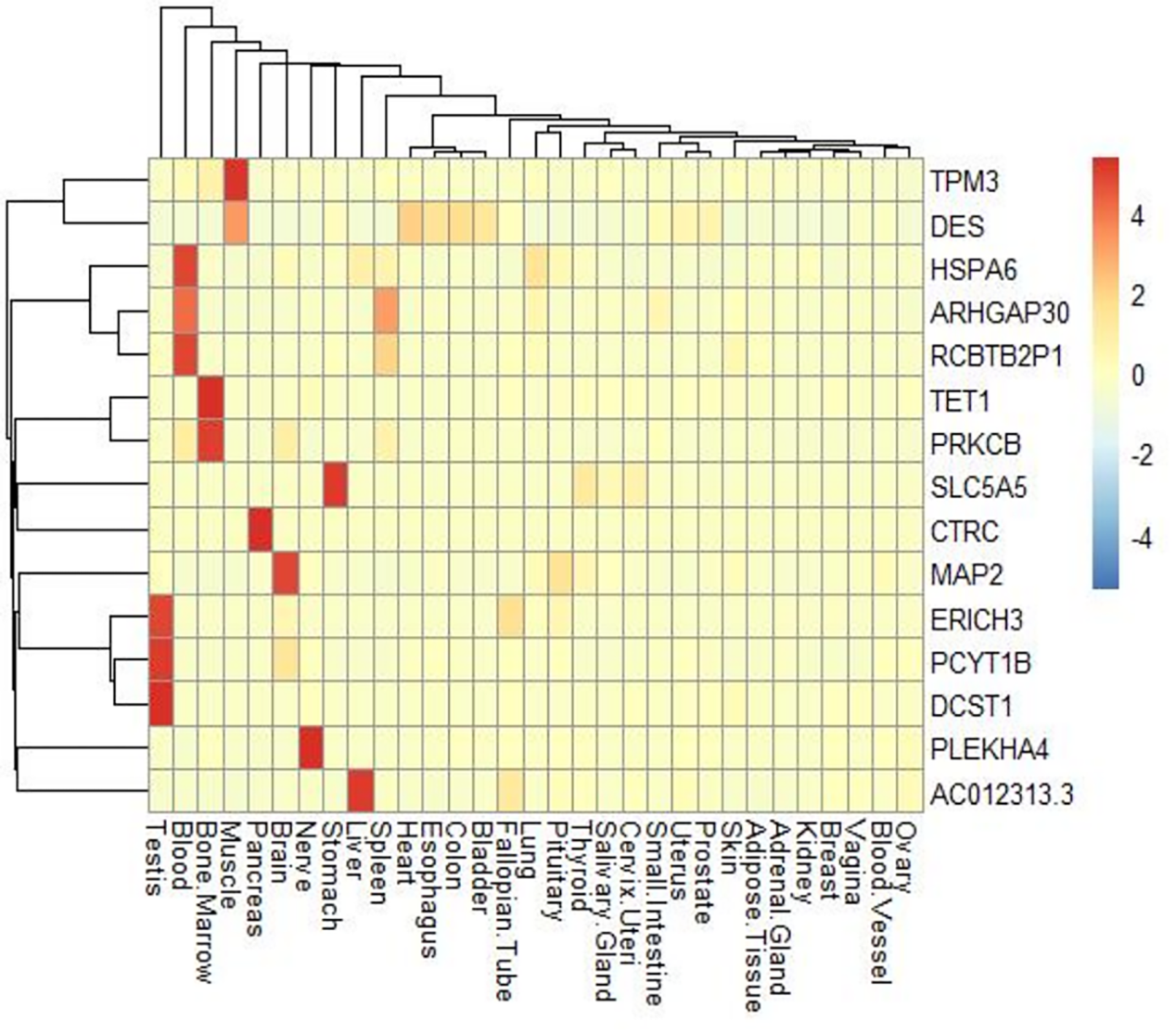


**Supplementary Figure 3. The heatmap shows the log 2FC value of tissue specific genes with S≥1.**Data of 31 human tissues, covering the expression levels of 60498 genes were downloaded from the GTEX database. S: the tissue specificity score. GTEX: Genotype-Tissue Expression.

**Supplementary Table 1. Laboratory Test Results of Enrolled Patients**

| **Groups** | **All**  **(n=20)** | **Control group (n=7)** | **NS group**  **( n=13)** | P-value |
| --- | --- | --- | --- | --- |
| Alanine aminotransferase, mean ± SD, (U/L) | 21.63±14.19 | 16.5±2.95 | 24±16.70 | 0.14 |
| Lactic dehydrogenase mean ± SD, (U/L) | 312.05±78.59 | 324.33±40.29 | 306.38±92.07 | 0.66 |
| Creatine kinase, mean ± SD, (U/L) | 246.84±461.75 | 149.83±112.90 | 291.62±554.65 | 0.55 |
| Creatine Kinase Isoenzyme, mean ± SD, (U/L) | 30.74±11.56 | 30.17±5.00 | 31±13.78 | 0.85 |
| CSF WBC (10^6/L)  (Increased, n, %) | 50.85±92.15 | 47.57±115.80  (1, 14.29%) | 52.62±82.05  (5, 38.46%) | 0.91 |
| CSF Hs-CRP, mean ± SD, (mg/L) | 0.23±0.16 | 0.27±0.20 | 0.21±0.14 | 0.48 |
| CSF Glucose, (mmol/L) | 3.47±1.15 | 3.68±0.86 | 3.36±1.30 | 0.57 |
| CSF chlorinum mean ± SD, (mmol/L) | 121.88±3.94 | 123.14±2.85 | 121.19±4.37 | 0.30 |
| Prothrombin time, mean ± SD, (s) | 13.78±0.89 | 13.47±0.66 | 13.95±0.98 | 0.26 |
| Activated partial thromboplastin time, mean ± SD, (s) | 41.57±6.88 | 40.6±5.78 | 42.08±7.58 | 0.66 |
| Fibrinogen, mean ± SD, (g/L) | 3.91±1.04 | 3.67±1.07 | 4.04±1.04 | 0.45 |
| International normalized ratio, mean ± SD | 1.06±0.09 | 1.02±0.06 | 1.07±0.10 | 0.22 |
| Monocytes, mean ± SD (10^9/L) | 0.83±0.55 | 0.73±0.23 | 0.88±0.66 | 0.59 |
| Neutrophils, mean±SD (10^9/L) | 6.07±2.31 | 5.60±2.55 | 6.32±2.24 | 0.52 |
| Blood platelet, mean±SD (10^9/L) | 326.80±136.18 | 299.86±117.72 | 341.31±147.58 | 0.53 |
| White blood cell, mean±SD (10^9/L) | 10.40±2.80 | 10.83±2.45 | 10.16±3.05 | 0.62 |
| Sodium, mean±SD (mmol/L) | 132.12±3.12 | 132.83±1.94 | 131.77±3.59 | 0.51 |
| Lactic acid, mean±SD (mmol/L) | 1.73±0.72 | 1.47±0.53 | 1.86±0.78 | 0.28 |
| Immunoglobulin A, mean±SD (g/L) | 0.28±0.29 | 0.29±0.18 | 0.28±0.34 | 0.97 |
| Immunoglobulin G, mean±SD (g/L) | 6.24±3.21 | 6.83±2.49 | 5.94±3.58 | 0.60 |
| Immunoglobulin M, mean±SD (g/L) | 0.97±0.82 | 1.07±0.69 | 0.93±0.90 | 0.74 |
| Complement C3, mean±SD (g/L) | 0.98±0.21 | 0.98±0.22 | 0.98±0.21 | 0.99 |
| Complement C4, mean±SD (g/L) | 0.23±0.08 | 0.23±0.07 | 0.24±0.09 | 0.83 |

**Supplementary Table 2. All Differentially-expressed Input RNA Genes**

| Gene Name | Gene ID | Regulation | Gene Biotype | log2FC | FDR | Pvalue |
| --- | --- | --- | --- | --- | --- | --- |
| MT-RNR2 | ENSG00000210082 | up | Mt_rRNA | 1.793355 | 0 | 0 |
| MT-ATP6 | ENSG00000198899 | down | protein_coding | -4.5763 | 0 | 0 |
| RPS23 | ENSG00000186468 | down | protein_coding | -5.76759 | 0 | 0 |
| RPS15 | ENSG00000115268 | down | protein_coding | -9.8552 | 0 | 0 |
| MT-CO2 | ENSG00000198712 | down | protein_coding | -9.67509 | 0 | 0 |
| EMP3 | ENSG00000142227 | down | protein_coding | -6.56238 | 0 | 0 |
| HSP90AB1 | ENSG00000096384 | down | protein_coding | -4.73739 | 0 | 0 |
| HSP90AA1 | ENSG00000080824 | down | protein_coding | -3.40525 | 0 | 0 |
| MAP4 | ENSG00000047849 | down | protein_coding | -10.943 | 0 | 0 |
| CASC3 | ENSG00000108349 | down | protein_coding | -4.70761 | 0 | 0 |
| ENO1 | ENSG00000074800 | down | protein_coding | -10.1362 | 0 | 0 |
| BCL2L1 | ENSG00000171552 | down | protein_coding | -10.8885 | 0 | 0 |
| PTK2 | ENSG00000169398 | down | protein_coding | -6.65357 | 0 | 0 |
| MT-ND2 | ENSG00000198763 | up | protein_coding | 4.865544 | 0 | 0 |
| EIF3B | ENSG00000106263 | down | protein_coding | -5.37944 | 1.41E-299 | 1.84E-301 |
| STIP1 | ENSG00000168439 | down | protein_coding | -6.3886 | 1.33E-299 | 1.85E-301 |
| AMOTL1 | ENSG00000166025 | down | protein_coding | -9.79476 | 5.09E-275 | 7.53E-277 |
| FILIP1L | ENSG00000168386 | down | protein_coding | -13.0964 | 2.56E-257 | 4.01E-259 |
| JUNB | ENSG00000171223 | down | protein_coding | -13.0641 | 3.23E-253 | 5.34E-255 |
| RAB10 | ENSG00000084733 | down | protein_coding | -11.6809 | 9.90E-251 | 1.72E-252 |
| HNRNPA2B1 | ENSG00000122566 | up | protein_coding | 7.755649 | 9.73E-251 | 1.78E-252 |
| CENPE | ENSG00000138778 | down | protein_coding | -13.0017 | 1.63E-245 | 3.11E-247 |
| MALAT1 | ENSG00000251562 | down | lincRNA | -1.95433 | 1.75E-245 | 3.51E-247 |
| CSNK1A1 | ENSG00000113712 | down | protein_coding | -8.3203 | 2.61E-238 | 5.44E-240 |
| AK3 | ENSG00000147853 | down | protein_coding | -5.12794 | 9.18E-230 | 2.00E-231 |
| JCAD | ENSG00000165757 | down | protein_coding | -3.92485 | 1.02E-229 | 2.31E-231 |
| MED17 | ENSG00000042429 | down | protein_coding | -6.71255 | 2.97E-222 | 6.97E-224 |
| RABL6 | ENSG00000196642 | up | protein_coding | 6.373961 | 4.75E-210 | 1.16E-211 |
| AHNAK2 | ENSG00000185567 | down | protein_coding | -8.79476 | 2.40E-209 | 6.04E-211 |
| MT-RNR1 | ENSG00000211459 | up | Mt_rRNA | 1.958862 | 1.38E-205 | 3.59E-207 |
| GTF2F1 | ENSG00000125651 | down | protein_coding | -5.81726 | 4.02E-204 | 1.08E-205 |
| ERICH3 | ENSG00000178965 | up | protein_coding | 2.939358 | 1.32E-197 | 3.68E-199 |
| RN7SL4P | ENSG00000263740 | up | misc_RNA | 4.034779 | 9.52E-197 | 2.73E-198 |
| PAIP2 | ENSG00000120727 | down | protein_coding | -12.5382 | 1.06E-194 | 3.13E-196 |
| PNRC2 | ENSG00000189266 | down | protein_coding | -5.73826 | 2.51E-193 | 7.65E-195 |
| BAZ1B | ENSG00000009954 | down | protein_coding | -12.4769 | 8.65E-189 | 2.71E-190 |
| FECH | ENSG00000066926 | down | protein_coding | -12.3987 | 1.74E-181 | 5.60E-183 |
| SEC11A | ENSG00000140612 | down | protein_coding | -11.0385 | 1.39E-179 | 4.58E-181 |
| PHB | ENSG00000167085 | down | protein_coding | -12.3556 | 1.38E-177 | 4.70E-179 |
| KIAA0368 | ENSG00000136813 | down | protein_coding | -10.9798 | 3.90E-174 | 1.36E-175 |
| ZSCAN26 | ENSG00000197062 | down | protein_coding | -12.2626 | 2.00E-169 | 7.12E-171 |
| YAP1 | ENSG00000137693 | down | protein_coding | -12.2259 | 2.68E-166 | 9.78E-168 |
| EGR1 | ENSG00000120738 | down | protein_coding | -10.8852 | 1.09E-165 | 4.09E-167 |
| RBPJL | ENSG00000124232 | down | protein_coding | -12.1521 | 3.39E-160 | 1.30E-161 |
| FTO | ENSG00000140718 | down | protein_coding | -4.28979 | 7.49E-160 | 2.93E-161 |
| IGF2 | ENSG00000167244 | down | protein_coding | -2.31441 | 8.88E-160 | 3.55E-161 |
| AC005224.3 | ENSG00000266378 | up | lincRNA | 8.126587 | 5.74E-157 | 2.34E-158 |
| ANP32A | ENSG00000140350 | down | protein_coding | -12.0862 | 6.08E-155 | 2.54E-156 |
| FURIN | ENSG00000140564 | down | protein_coding | -12.0172 | 1.39E-149 | 5.91E-151 |
| AGO1 | ENSG00000092847 | down | protein_coding | -11.9955 | 6.06E-148 | 2.63E-149 |
| DDB1 | ENSG00000167986 | down | protein_coding | -11.9798 | 9.04E-147 | 4.01E-148 |
| TPM3 | ENSG00000143549 | down | protein_coding | -5.31363 | 2.22E-143 | 1.00E-144 |
| MT-CO1 | ENSG00000198804 | up | protein_coding | 5.478344 | 1.30E-142 | 5.98E-144 |
| CSRNP1 | ENSG00000144655 | down | protein_coding | -4.64414 | 1.76E-142 | 8.28E-144 |
| NUPR1 | ENSG00000176046 | up | protein_coding | 9.132416 | 1.43E-140 | 6.82E-142 |
| MYH9 | ENSG00000100345 | up | protein_coding | 5.735875 | 8.75E-137 | 4.26E-138 |
| NBEA | ENSG00000172915 | down | protein_coding | -11.8407 | 9.98E-137 | 4.94E-138 |
| KDM6A | ENSG00000147050 | up | protein_coding | 9.052279 | 2.19E-134 | 1.11E-135 |
| AQP3 | ENSG00000165272 | down | protein_coding | -11.7948 | 1.47E-133 | 7.55E-135 |
| ADCY6 | ENSG00000174233 | down | protein_coding | -11.7694 | 7.73E-132 | 4.03E-133 |
| PRDX1 | ENSG00000117450 | up | protein_coding | 9.013639 | 1.67E-131 | 8.84E-133 |
| ZC3H18 | ENSG00000158545 | up | protein_coding | 8.988149 | 1.25E-129 | 6.74E-131 |
| PBDC1 | ENSG00000102390 | up | protein_coding | 5.653953 | 2.85E-129 | 1.56E-130 |
| GFM1 | ENSG00000168827 | up | protein_coding | 7.789983 | 1.35E-128 | 7.53E-130 |
| CAPN2 | ENSG00000162909 | up | protein_coding | 7.773664 | 2.39E-127 | 1.35E-128 |
| PNPT1 | ENSG00000138035 | down | protein_coding | -11.6751 | 1.23E-125 | 7.07E-127 |
| POU2F1 | ENSG00000143190 | down | protein_coding | -11.6475 | 7.15E-124 | 4.17E-125 |
| CMTR2 | ENSG00000180917 | down | protein_coding | -11.6396 | 2.27E-123 | 1.34E-124 |
| FAM32A | ENSG00000105058 | up | protein_coding | 7.687599 | 6.03E-121 | 3.62E-122 |
| ILF3 | ENSG00000129351 | up | protein_coding | 7.665256 | 2.47E-119 | 1.51E-120 |
| NPHP1 | ENSG00000144061 | down | protein_coding | -10.247 | 4.47E-118 | 2.76E-119 |
| HSP90B1 | ENSG00000166598 | up | protein_coding | 3.00095 | 1.85E-116 | 1.16E-117 |
| PDE6A | ENSG00000132915 | down | protein_coding | -2.56361 | 9.30E-115 | 5.90E-116 |
| ANP32B | ENSG00000136938 | up | protein_coding | 5.85253 | 1.93E-111 | 1.24E-112 |
| TCEAL8 | ENSG00000180964 | up | protein_coding | 8.708041 | 1.33E-110 | 8.65E-112 |
| TXNIP | ENSG00000265972 | down | protein_coding | -11.4198 | 3.53E-110 | 2.35E-111 |
| UEVLD | ENSG00000151116 | down | protein_coding | -11.4198 | 3.53E-110 | 2.35E-111 |
| RPS20 | ENSG00000008988 | up | protein_coding | 7.51985 | 2.59E-109 | 1.76E-110 |
| C22orf46 | ENSG00000184208 | up | protein_coding | 8.678066 | 9.60E-109 | 6.59E-110 |
| TYMP | ENSG00000025708 | down | protein_coding | -11.3773 | 8.49E-108 | 5.91E-109 |
| CTNNBL1 | ENSG00000132792 | up | protein_coding | 8.660423 | 1.16E-107 | 8.19E-109 |
| FTL | ENSG00000087086 | up | protein_coding | 7.487469 | 3.35E-107 | 2.39E-108 |
| UPP1 | ENSG00000183696 | up | protein_coding | 8.636011 | 3.56E-106 | 2.57E-107 |
| PGGT1B | ENSG00000164219 | up | protein_coding | 7.46916 | 5.06E-106 | 3.70E-107 |
| MICU2 | ENSG00000165487 | up | protein_coding | 8.621163 | 2.77E-105 | 2.05E-106 |
| ZNF337 | ENSG00000130684 | up | protein_coding | 8.587611 | 2.80E-103 | 2.10E-104 |
| GAPDH | ENSG00000111640 | up | protein_coding | 5.305441 | 3.65E-101 | 2.76E-102 |
| SYF2 | ENSG00000117614 | up | protein_coding | 8.54629 | 7.28E-101 | 5.60E-102 |
| TAT | ENSG00000198650 | up | protein_coding | 8.54629 | 7.28E-101 | 5.60E-102 |
| HP | ENSG00000257017 | down | protein_coding | -11.247 | 8.59E-101 | 6.73E-102 |
| TACC3 | ENSG00000013810 | down | protein_coding | -11.2365 | 3.02E-100 | 2.39E-101 |
| MAP2K5 | ENSG00000137764 | up | protein_coding | 8.516288 | 3.75E-99 | 3.00E-100 |
| ZMIZ1 | ENSG00000108175 | up | protein_coding | 8.514503 | 4.69E-99 | 3.79E-100 |
| INTS8 | ENSG00000164941 | down | protein_coding | -9.87842 | 7.86E-97 | 6.43E-98 |
| COX20 | ENSG00000203667 | down | protein_coding | -11.1267 | 1.08E-94 | 8.92E-96 |
| AC005759.1 | ENSG00000268650 | down | antisense_RNA | -11.1153 | 3.91E-94 | 3.27E-95 |
| ITIH2 | ENSG00000151655 | down | protein_coding | -11.1095 | 7.42E-94 | 6.26E-95 |
| YOD1 | ENSG00000180667 | up | protein_coding | 8.410881 | 2.55E-93 | 2.17E-94 |
| PLEC | ENSG00000178209 | up | protein_coding | 6.182721 | 3.32E-93 | 2.86E-94 |
| ZNF473 | ENSG00000142528 | down | protein_coding | -11.0921 | 5.09E-93 | 4.43E-94 |
| RPL19 | ENSG00000108298 | up | protein_coding | 5.568246 | 1.11E-91 | 9.82E-93 |
| UBXN1 | ENSG00000162191 | up | protein_coding | 8.362103 | 9.69E-91 | 8.68E-92 |
| DDX21 | ENSG00000165732 | up | protein_coding | 6.141694 | 1.02E-90 | 9.22E-92 |
| NCK2 | ENSG00000071051 | up | protein_coding | 8.344132 | 8.31E-90 | 7.59E-91 |
| SNRNP70 | ENSG00000104852 | up | protein_coding | 8.33405 | 2.75E-89 | 2.54E-90 |
| COX6C | ENSG00000164919 | up | protein_coding | 8.319816 | 1.48E-88 | 1.38E-89 |
| MRM1 | ENSG00000278619 | up | protein_coding | 8.272034 | 3.94E-86 | 3.70E-87 |
| FCN3 | ENSG00000142748 | up | protein_coding | 8.261433 | 1.32E-85 | 1.25E-86 |
| SPAG9 | ENSG00000008294 | down | protein_coding | -9.6515 | 1.32E-85 | 1.26E-86 |
| NRAS | ENSG00000213281 | up | protein_coding | 8.252897 | 3.45E-85 | 3.33E-86 |
| SERBP1 | ENSG00000142864 | up | protein_coding | 8.242154 | 1.17E-84 | 1.13E-85 |
| NUFIP2 | ENSG00000108256 | up | protein_coding | 7.053509 | 3.60E-82 | 3.53E-83 |
| PSIP1 | ENSG00000164985 | up | protein_coding | 4.714273 | 1.27E-81 | 1.26E-82 |
| SLC35C1 | ENSG00000181830 | up | protein_coding | 8.144004 | 5.95E-80 | 5.95E-81 |
| RTL5 | ENSG00000242732 | down | protein_coding | -10.7876 | 2.33E-79 | 2.35E-80 |
| GLUL | ENSG00000135821 | up | protein_coding | 8.127755 | 3.34E-79 | 3.40E-80 |
| G3BP2 | ENSG00000138757 | up | protein_coding | 8.120734 | 7.01E-79 | 7.19E-80 |
| SLC40A1 | ENSG00000138449 | up | protein_coding | 8.11368 | 1.47E-78 | 1.52E-79 |
| RPL28 | ENSG00000108107 | up | protein_coding | 8.097083 | 8.42E-78 | 8.78E-79 |
| SNX33 | ENSG00000173548 | up | protein_coding | 8.092306 | 1.38E-77 | 1.45E-78 |
| GLI1 | ENSG00000111087 | up | protein_coding | 8.080294 | 4.80E-77 | 5.09E-78 |
| BUD13 | ENSG00000137656 | up | protein_coding | 8.060863 | 3.56E-76 | 3.81E-77 |
| NFKBIA | ENSG00000100906 | up | protein_coding | 8.058416 | 4.53E-76 | 4.90E-77 |
| INTS7 | ENSG00000143493 | up | protein_coding | 8.058416 | 4.53E-76 | 4.90E-77 |
| RPS4Y1 | ENSG00000129824 | down | protein_coding | -10.6829 | 3.89E-75 | 4.26E-76 |
| NUBP1 | ENSG00000103274 | up | protein_coding | 8.02371 | 1.53E-74 | 1.69E-75 |
| ITGAM | ENSG00000169896 | down | protein_coding | -10.6673 | 1.58E-74 | 1.76E-75 |
| PPM1G | ENSG00000115241 | up | protein_coding | 8.021199 | 1.94E-74 | 2.18E-75 |
| FKBP5 | ENSG00000096060 | up | protein_coding | 5.842134 | 3.06E-74 | 3.46E-75 |
| LRPPRC | ENSG00000138095 | up | protein_coding | 8.01111 | 5.27E-74 | 6.00E-75 |
| RPL18 | ENSG00000063177 | up | protein_coding | 7.998399 | 1.86E-73 | 2.14E-74 |
| ACAT1 | ENSG00000075239 | up | protein_coding | 5.824959 | 2.18E-73 | 2.52E-74 |
| APOE | ENSG00000130203 | up | protein_coding | 7.982996 | 8.47E-73 | 9.86E-74 |
| WSB1 | ENSG00000109046 | up | protein_coding | 7.977825 | 1.40E-72 | 1.64E-73 |
| WDR60 | ENSG00000126870 | down | protein_coding | -10.6113 | 2.19E-72 | 2.60E-73 |
| CLEC4D | ENSG00000166527 | up | protein_coding | 7.95958 | 8.25E-72 | 9.83E-73 |
| BCAR3 | ENSG00000137936 | down | protein_coding | -10.5949 | 9.10E-72 | 1.09E-72 |
| KIAA1147 | ENSG00000257093 | up | protein_coding | 7.954324 | 1.36E-71 | 1.64E-72 |
| APOB | ENSG00000084674 | down | protein_coding | -10.5783 | 3.79E-71 | 4.62E-72 |
| YWHAE | ENSG00000108953 | down | protein_coding | -7.42911 | 1.05E-70 | 1.29E-71 |
| SWAP70 | ENSG00000133789 | up | protein_coding | 7.900686 | 2.27E-69 | 2.81E-70 |
| IGF1R | ENSG00000140443 | up | protein_coding | 7.861916 | 8.48E-68 | 1.05E-68 |
| HNRNPU | ENSG00000153187 | up | protein_coding | 4.245744 | 8.66E-68 | 1.08E-68 |
| COL4A1 | ENSG00000187498 | up | protein_coding | 7.853471 | 1.82E-67 | 2.30E-68 |
| PABPC1 | ENSG00000070756 | down | protein_coding | -1.90225 | 1.08E-66 | 1.37E-67 |
| CEP250 | ENSG00000126001 | up | protein_coding | 6.704915 | 4.39E-66 | 5.61E-67 |
| B2M | ENSG00000166710 | up | protein_coding | 6.698642 | 7.99E-66 | 1.03E-66 |
| PFDN5 | ENSG00000123349 | up | protein_coding | 7.801736 | 1.95E-65 | 2.52E-66 |
| KIF5B | ENSG00000170759 | up | protein_coding | 4.42006 | 4.33E-65 | 5.65E-66 |
| TAF15 | ENSG00000270647 | up | protein_coding | 5.085111 | 4.39E-65 | 5.77E-66 |
| MTSS1L | ENSG00000132613 | up | protein_coding | 7.787029 | 7.08E-65 | 9.35E-66 |
| BTG2 | ENSG00000159388 | up | protein_coding | 7.748076 | 2.16E-63 | 2.87E-64 |
| PEG10 | ENSG00000242265 | up | protein_coding | 7.729736 | 1.05E-62 | 1.40E-63 |
| CDYL | ENSG00000153046 | up | protein_coding | 7.717379 | 3.00E-62 | 4.04E-63 |
| C1orf115 | ENSG00000162817 | up | protein_coding | 7.71116 | 5.07E-62 | 6.87E-63 |
| TC2N | ENSG00000165929 | down | protein_coding | -10.3136 | 7.08E-62 | 9.67E-63 |
| PRKCB | ENSG00000166501 | up | protein_coding | 7.701782 | 1.11E-61 | 1.53E-62 |
| MCM7 | ENSG00000166508 | down | protein_coding | -10.3036 | 1.50E-61 | 2.07E-62 |
| ALDOA | ENSG00000149925 | up | protein_coding | 7.695496 | 1.87E-61 | 2.60E-62 |
| YBX3 | ENSG00000060138 | down | protein_coding | -9.03853 | 4.05E-61 | 5.67E-62 |
| UPF1 | ENSG00000005007 | up | protein_coding | 7.686015 | 4.10E-61 | 5.77E-62 |
| ZEB2 | ENSG00000169554 | up | protein_coding | 7.68284 | 5.31E-61 | 7.53E-62 |
| KCTD3 | ENSG00000136636 | down | protein_coding | -10.2626 | 3.08E-60 | 4.39E-61 |
| PLA2G16 | ENSG00000176485 | up | protein_coding | 7.657192 | 4.45E-60 | 6.38E-61 |
| RPL5 | ENSG00000122406 | up | protein_coding | 7.653953 | 5.77E-60 | 8.34E-61 |
| THAP5 | ENSG00000177683 | down | protein_coding | -10.2522 | 6.52E-60 | 9.46E-61 |
| TAX1BP1 | ENSG00000106052 | up | protein_coding | 7.650708 | 7.46E-60 | 1.09E-60 |
| AIMP1 | ENSG00000164022 | up | protein_coding | 7.647455 | 9.69E-60 | 1.42E-60 |
| ZNF274 | ENSG00000171606 | up | protein_coding | 7.640927 | 1.65E-59 | 2.43E-60 |
| RN7SL752P | ENSG00000239437 | up | misc_RNA | 6.510928 | 1.93E-58 | 2.88E-59 |
| NAGK | ENSG00000124357 | up | protein_coding | 7.607836 | 2.39E-58 | 3.58E-59 |
| AMMECR1L | ENSG00000144233 | up | protein_coding | 7.594384 | 6.98E-58 | 1.05E-58 |
| HIST1H2BC | ENSG00000180596 | up | protein_coding | 7.594384 | 6.98E-58 | 1.05E-58 |
| RPS3 | ENSG00000149273 | up | protein_coding | 4.907954 | 3.38E-57 | 5.14E-58 |
| GMDS-AS1 | ENSG00000250903 | up | lincRNA | 7.560195 | 1.03E-56 | 1.58E-57 |
| RN7SKP219 | ENSG00000252634 | up | misc_RNA | 7.556732 | 1.34E-56 | 2.07E-57 |
| UBC | ENSG00000150991 | up | protein_coding | 7.525177 | 1.54E-55 | 2.39E-56 |
| UBB | ENSG00000170315 | up | protein_coding | 7.50375 | 7.90E-55 | 1.23E-55 |
| MAFK | ENSG00000198517 | up | protein_coding | 7.463622 | 1.60E-53 | 2.51E-54 |
| VIM | ENSG00000026025 | up | protein_coding | 7.459918 | 2.10E-53 | 3.30E-54 |
| ZNF267 | ENSG00000185947 | down | protein_coding | -10.0264 | 3.89E-53 | 6.16E-54 |
| VPS13C | ENSG00000129003 | up | protein_coding | 7.448749 | 4.74E-53 | 7.55E-54 |
| NFIC | ENSG00000141905 | down | protein_coding | -8.75842 | 2.80E-52 | 4.48E-53 |
| RINT1 | ENSG00000135249 | up | protein_coding | 7.418534 | 4.27E-52 | 6.87E-53 |
| NEAT1 | ENSG00000245532 | up | lincRNA | 4.775155 | 7.89E-52 | 1.28E-52 |
| AD000090.1 | ENSG00000283907 | up | antisense_RNA | 7.399324 | 1.69E-51 | 2.75E-52 |
| MT-ND6 | ENSG00000198695 | up | protein_coding | 4.104219 | 1.31E-50 | 2.15E-51 |
| CDC5L | ENSG00000096401 | up | protein_coding | 7.364087 | 2.04E-50 | 3.36E-51 |
| RCC2 | ENSG00000179051 | up | protein_coding | 7.336072 | 1.43E-49 | 2.37E-50 |
| TIPARP | ENSG00000163659 | up | protein_coding | 7.327967 | 2.50E-49 | 4.15E-50 |
| FP671120.4 | ENSG00000281383 | up | lincRNA | 7.311619 | 7.63E-49 | 1.27E-49 |
| HUWE1 | ENSG00000086758 | up | protein_coding | 4.693394 | 9.53E-49 | 1.60E-49 |
| MTUS1 | ENSG00000129422 | up | protein_coding | 7.303375 | 1.32E-48 | 2.24E-49 |
| MED15 | ENSG00000099917 | up | protein_coding | 7.303375 | 1.32E-48 | 2.24E-49 |
| COMMD6 | ENSG00000188243 | up | protein_coding | 7.286744 | 4.04E-48 | 6.89E-49 |
| ZBTB38 | ENSG00000177311 | up | protein_coding | 7.282557 | 5.32E-48 | 9.13E-49 |
| CDR1 | ENSG00000184258 | up | protein_coding | 7.282557 | 5.32E-48 | 9.13E-49 |
| ZNF224 | ENSG00000267680 | up | protein_coding | 7.265683 | 1.63E-47 | 2.82E-48 |
| MT-ND5 | ENSG00000198786 | down | protein_coding | -5.33353 | 1.83E-47 | 3.19E-48 |
| CCNI | ENSG00000118816 | up | protein_coding | 1.934079 | 8.62E-47 | 1.51E-47 |
| PTMA | ENSG00000187514 | up | protein_coding | 4.006039 | 1.03E-46 | 1.82E-47 |
| IFITM2 | ENSG00000185201 | up | protein_coding | 7.231331 | 1.54E-46 | 2.72E-47 |
| BLCAP | ENSG00000166619 | down | protein_coding | -9.75842 | 4.70E-46 | 8.33E-47 |
| MBD5 | ENSG00000204406 | up | protein_coding | 7.213844 | 4.76E-46 | 8.48E-47 |
| CAST | ENSG00000153113 | up | protein_coding | 6.132416 | 9.19E-46 | 1.65E-46 |
| DECR1 | ENSG00000104325 | down | protein_coding | -9.6983 | 1.37E-44 | 2.46E-45 |
| SATB1-AS1 | ENSG00000228956 | up | processed_transcript | 7.11368 | 2.56E-43 | 4.62E-44 |
| PRKCD | ENSG00000163932 | up | protein_coding | 7.07546 | 2.57E-42 | 4.67E-43 |
| WTAP | ENSG00000146457 | up | protein_coding | 7.055964 | 8.14E-42 | 1.49E-42 |
| BAZ1A | ENSG00000198604 | up | protein_coding | 7.055964 | 8.14E-42 | 1.49E-42 |
| EPRS | ENSG00000136628 | up | protein_coding | 7.051049 | 1.08E-41 | 1.99E-42 |
| CRYZL1 | ENSG00000205758 | up | protein_coding | 7.031218 | 3.44E-41 | 6.38E-42 |
| RPL26L1 | ENSG00000037241 | up | protein_coding | 7.031218 | 3.44E-41 | 6.38E-42 |
| KDM2B | ENSG00000089094 | up | protein_coding | 6.995843 | 2.64E-40 | 4.93E-41 |
| FMNL2 | ENSG00000157827 | down | protein_coding | -3.43744 | 8.31E-40 | 1.56E-40 |
| SOD2 | ENSG00000112096 | up | protein_coding | 6.970034 | 1.13E-39 | 2.14E-40 |
| ITGB2 | ENSG00000160255 | up | protein_coding | 6.970034 | 1.13E-39 | 2.14E-40 |
| EEF1A1 | ENSG00000156508 | up | protein_coding | 4.421077 | 1.33E-39 | 2.54E-40 |
| FCN1 | ENSG00000085265 | up | protein_coding | 6.949049 | 3.62E-39 | 6.93E-40 |
| ZDHHC24 | ENSG00000174165 | up | protein_coding | 6.922381 | 1.58E-38 | 3.03E-39 |
| ST3GAL1 | ENSG00000008513 | up | protein_coding | 6.900686 | 5.12E-38 | 9.89E-39 |
| STAT6 | ENSG00000166888 | down | protein_coding | -9.39164 | 8.69E-38 | 1.68E-38 |
| AL691432.2 | ENSG00000272106 | down | antisense_RNA | -9.37253 | 2.13E-37 | 4.15E-38 |
| HNRNPC | ENSG00000092199 | up | protein_coding | 6.867519 | 3.00E-37 | 5.88E-38 |
| AL158835.1 | ENSG00000228021 | up | processed_transcript | 6.861916 | 4.03E-37 | 7.91E-38 |
| ALB | ENSG00000163631 | down | protein_coding | -9.35316 | 5.19E-37 | 1.02E-37 |
| TET3 | ENSG00000187605 | up | protein_coding | 5.810488 | 5.49E-37 | 1.09E-37 |
| ACTN4 | ENSG00000130402 | up | protein_coding | 2.210909 | 5.95E-37 | 1.18E-37 |
| PLEKHA4 | ENSG00000105559 | up | protein_coding | 6.850645 | 7.18E-37 | 1.44E-37 |
| RNF145 | ENSG00000145860 | down | protein_coding | -9.33353 | 1.26E-36 | 2.54E-37 |
| PXN | ENSG00000089159 | down | protein_coding | -9.31363 | 3.13E-36 | 6.30E-37 |
| TINAGL1 | ENSG00000142910 | up | protein_coding | 6.798806 | 1.05E-35 | 2.12E-36 |
| TBC1D31 | ENSG00000156787 | down | protein_coding | -8.07445 | 1.79E-35 | 3.64E-36 |
| COPG1 | ENSG00000181789 | up | protein_coding | 6.775155 | 3.45E-35 | 7.06E-36 |
| FAU | ENSG00000149806 | up | protein_coding | 6.769181 | 4.65E-35 | 9.54E-36 |
| SCARNA6 | ENSG00000251791 | up | scaRNA | 6.726657 | 3.83E-34 | 7.90E-35 |
| JAK3 | ENSG00000105639 | up | protein_coding | 6.708041 | 9.48E-34 | 1.96E-34 |
| CDK4 | ENSG00000135446 | up | protein_coding | 6.670073 | 5.86E-33 | 1.22E-33 |
| ASXL2 | ENSG00000143970 | down | protein_coding | -6.2026 | 6.50E-33 | 1.36E-33 |
| RN7SL3 | ENSG00000278771 | up | misc_RNA | 5.604485 | 3.38E-32 | 7.09E-33 |
| CHMP2A | ENSG00000130724 | up | protein_coding | 6.597759 | 1.68E-31 | 3.53E-32 |
| RAD17 | ENSG00000152942 | down | protein_coding | -9.0506 | 2.16E-31 | 4.57E-32 |
| TPT1 | ENSG00000133112 | up | protein_coding | 5.563651 | 2.56E-31 | 5.44E-32 |
| DNAJA1 | ENSG00000086061 | up | protein_coding | 6.584212 | 3.07E-31 | 6.54E-32 |
| MIB1 | ENSG00000101752 | down | protein_coding | -9.02635 | 5.56E-31 | 1.19E-31 |
| EPSTI1 | ENSG00000133106 | up | protein_coding | 6.556732 | 1.05E-30 | 2.25E-31 |
| RAP1GDS1 | ENSG00000138698 | down | protein_coding | -7.81613 | 1.46E-30 | 3.14E-31 |
| GGCX | ENSG00000115486 | down | protein_coding | -8.95106 | 9.93E-30 | 2.15E-30 |
| PFN1 | ENSG00000108518 | up | protein_coding | 4.549779 | 1.51E-29 | 3.28E-30 |
| GIMAP4 | ENSG00000133574 | up | protein_coding | 6.492917 | 1.68E-29 | 3.66E-30 |
| ZSWIM7 | ENSG00000214941 | up | protein_coding | 6.485649 | 2.28E-29 | 5.00E-30 |
| CBFB | ENSG00000067955 | up | protein_coding | 6.471002 | 4.24E-29 | 9.34E-30 |
| CALD1 | ENSG00000122786 | up | protein_coding | 6.463622 | 5.78E-29 | 1.28E-29 |
| RETREG3 | ENSG00000141699 | down | protein_coding | -8.8986 | 6.82E-29 | 1.51E-29 |
| KIF1C | ENSG00000129250 | up | protein_coding | 6.441254 | 1.46E-28 | 3.26E-29 |
| PRPF19 | ENSG00000110107 | up | protein_coding | 6.426147 | 2.73E-28 | 6.10E-29 |
| STAU1 | ENSG00000124214 | down | protein_coding | -7.66727 | 4.92E-28 | 1.10E-28 |
| TCEAL9 | ENSG00000185222 | down | protein_coding | -4.76262 | 1.09E-27 | 2.46E-28 |
| PLCG1 | ENSG00000124181 | down | protein_coding | -8.81613 | 1.29E-27 | 2.92E-28 |
| MKKS | ENSG00000125863 | up | protein_coding | 6.364087 | 3.34E-27 | 7.58E-28 |
| RPL36AL | ENSG00000165502 | up | protein_coding | 6.348145 | 6.26E-27 | 1.43E-27 |
| FGA | ENSG00000171560 | down | protein_coding | -8.75842 | 9.41E-27 | 2.15E-27 |
| WARS | ENSG00000140105 | up | protein_coding | 6.332025 | 1.17E-26 | 2.69E-27 |
| FOXC1 | ENSG00000054598 | up | protein_coding | 6.323897 | 1.60E-26 | 3.69E-27 |
| NDUFV3 | ENSG00000160194 | up | protein_coding | 6.323897 | 1.60E-26 | 3.69E-27 |
| TLK1 | ENSG00000198586 | up | protein_coding | 6.315724 | 2.18E-26 | 5.07E-27 |
| ADGRD1 | ENSG00000111452 | up | protein_coding | 6.307503 | 2.99E-26 | 6.97E-27 |
| USP25 | ENSG00000155313 | up | protein_coding | 6.29092 | 5.63E-26 | 1.32E-26 |
| FADS1 | ENSG00000149485 | down | protein_coding | -6.42911 | 9.00E-26 | 2.11E-26 |
| NPM1 | ENSG00000181163 | up | protein_coding | 3.871242 | 1.54E-25 | 3.62E-26 |
| FYN | ENSG00000010810 | up | protein_coding | 6.257171 | 1.99E-25 | 4.72E-26 |
| DIAPH1 | ENSG00000131504 | up | protein_coding | 6.257171 | 1.99E-25 | 4.72E-26 |
| DRAP1 | ENSG00000175550 | up | protein_coding | 6.239996 | 3.76E-25 | 8.95E-26 |
| ATF4 | ENSG00000128272 | up | protein_coding | 5.239996 | 4.35E-25 | 1.04E-25 |
| BIN2 | ENSG00000110934 | up | protein_coding | 6.222614 | 7.08E-25 | 1.70E-25 |
| SFPQ | ENSG00000116560 | up | protein_coding | 4.315724 | 9.98E-25 | 2.40E-25 |
| SRP14 | ENSG00000140319 | up | protein_coding | 6.196142 | 1.84E-24 | 4.46E-25 |
| STX3 | ENSG00000166900 | up | protein_coding | 6.187209 | 2.54E-24 | 6.15E-25 |
| GTF2E2 | ENSG00000197265 | down | protein_coding | -8.56997 | 3.99E-24 | 9.73E-25 |
| C1S | ENSG00000182326 | down | protein_coding | -8.56997 | 3.99E-24 | 9.73E-25 |
| MYOF | ENSG00000138119 | up | protein_coding | 6.160073 | 6.60E-24 | 1.62E-24 |
| N4BP2L2 | ENSG00000244754 | up | protein_coding | 6.141694 | 1.26E-23 | 3.09E-24 |
| MSN | ENSG00000147065 | up | protein_coding | 3.456205 | 1.31E-23 | 3.25E-24 |
| DYNLT3 | ENSG00000165169 | down | protein_coding | -8.50126 | 3.15E-23 | 7.81E-24 |
| TIMP3 | ENSG00000100234 | up | protein_coding | 6.11368 | 3.29E-23 | 8.18E-24 |
| HSPA5 | ENSG00000044574 | up | protein_coding | 6.104219 | 4.53E-23 | 1.13E-23 |
| YWHAB | ENSG00000166913 | up | protein_coding | 5.07546 | 2.29E-22 | 5.74E-23 |
| GRK2 | ENSG00000173020 | up | protein_coding | 6.055964 | 2.30E-22 | 5.77E-23 |
| EML4 | ENSG00000143924 | down | protein_coding | -8.42911 | 2.55E-22 | 6.42E-23 |
| ZNF217 | ENSG00000171940 | up | protein_coding | 6.046116 | 3.16E-22 | 8.00E-23 |
| UFM1 | ENSG00000120686 | up | protein_coding | 6.006039 | 1.17E-21 | 2.97E-22 |
| NAA20 | ENSG00000173418 | up | protein_coding | 5.995843 | 1.62E-21 | 4.12E-22 |
| TRIM32 | ENSG00000119401 | up | protein_coding | 5.985575 | 2.24E-21 | 5.72E-22 |
| SNX20 | ENSG00000167208 | up | protein_coding | 5.985575 | 2.24E-21 | 5.72E-22 |
| EMD | ENSG00000102119 | up | protein_coding | 5.975233 | 3.09E-21 | 7.96E-22 |
| UBE2L6 | ENSG00000156587 | up | protein_coding | 5.964816 | 4.28E-21 | 1.11E-21 |
| STUB1 | ENSG00000103266 | up | protein_coding | 5.964816 | 4.28E-21 | 1.11E-21 |
| PSMD2 | ENSG00000175166 | up | protein_coding | 4.964816 | 1.08E-20 | 2.81E-21 |
| MX1 | ENSG00000157601 | up | protein_coding | 5.889715 | 4.30E-20 | 1.12E-20 |
| KYNU | ENSG00000115919 | up | protein_coding | 4.922381 | 4.46E-20 | 1.17E-20 |
| TRIM2 | ENSG00000109654 | down | protein_coding | -8.23117 | 5.32E-20 | 1.40E-20 |
| EEF1D | ENSG00000104529 | up | protein_coding | 5.87866 | 5.93E-20 | 1.56E-20 |
| FRMD8 | ENSG00000126391 | up | protein_coding | 5.87866 | 5.93E-20 | 1.56E-20 |
| SEMA7A | ENSG00000138623 | down | protein_coding | -8.1881 | 1.58E-19 | 4.19E-20 |
| TNR | ENSG00000116147 | up | protein_coding | 5.822076 | 3.11E-19 | 8.28E-20 |
| CHD6 | ENSG00000124177 | down | protein_coding | -8.14371 | 4.74E-19 | 1.27E-19 |
| BST2 | ENSG00000130303 | up | protein_coding | 5.798806 | 6.03E-19 | 1.62E-19 |
| RPL22 | ENSG00000116251 | up | protein_coding | 5.798806 | 6.03E-19 | 1.62E-19 |
| AIFM2 | ENSG00000042286 | up | protein_coding | 5.726657 | 4.51E-18 | 1.22E-18 |
| LMNA | ENSG00000160789 | up | protein_coding | 5.726657 | 4.51E-18 | 1.22E-18 |
| GOLGA4 | ENSG00000144674 | up | protein_coding | 2.747063 | 8.60E-18 | 2.33E-18 |
| RUVBL2 | ENSG00000183207 | up | protein_coding | 4.738935 | 1.31E-17 | 3.57E-18 |
| AC126474.2 | ENSG00000280088 | up | TEC | 5.676471 | 1.72E-17 | 4.71E-18 |
| PI4K2B | ENSG00000038210 | up | protein_coding | 5.676471 | 1.72E-17 | 4.71E-18 |
| FLII | ENSG00000177731 | up | protein_coding | 5.650708 | 3.37E-17 | 9.28E-18 |
| DOK2 | ENSG00000147443 | up | protein_coding | 5.650708 | 3.37E-17 | 9.28E-18 |
| WDFY1 | ENSG00000085449 | down | protein_coding | -7.95106 | 4.14E-17 | 1.15E-17 |
| ZNF622 | ENSG00000173545 | up | protein_coding | 5.637652 | 4.70E-17 | 1.30E-17 |
| ACTB | ENSG00000075624 | up | protein_coding | 3.39027 | 5.45E-17 | 1.52E-17 |
| IQCE | ENSG00000106012 | up | protein_coding | 5.624476 | 6.56E-17 | 1.83E-17 |
| FKBP4 | ENSG00000004478 | up | protein_coding | 4.676471 | 7.73E-17 | 2.16E-17 |
| CD63 | ENSG00000135404 | up | protein_coding | 5.597759 | 1.29E-16 | 3.62E-17 |
| ZNFX1 | ENSG00000124201 | up | protein_coding | 5.584212 | 1.81E-16 | 5.10E-17 |
| SERF2 | ENSG00000140264 | up | protein_coding | 3.055964 | 1.96E-16 | 5.53E-17 |
| DHRS3 | ENSG00000162496 | up | protein_coding | 5.570537 | 2.53E-16 | 7.18E-17 |
| RAB13 | ENSG00000143545 | up | protein_coding | 4.624476 | 3.22E-16 | 9.17E-17 |
| HSPD1 | ENSG00000144381 | up | protein_coding | 5.556732 | 3.54E-16 | 1.01E-16 |
| AC005181.1 | ENSG00000271101 | down | processed_pseudogene | -7.84415 | 4.02E-16 | 1.15E-16 |
| PPCS | ENSG00000127125 | up | protein_coding | 5.542792 | 4.96E-16 | 1.42E-16 |
| EEF2 | ENSG00000167658 | up | protein_coding | 3.763182 | 5.85E-16 | 1.68E-16 |
| NFE2L1 | ENSG00000082641 | up | protein_coding | 5.528717 | 6.96E-16 | 2.01E-16 |
| METTL9 | ENSG00000197006 | up | protein_coding | 4.584212 | 9.36E-16 | 2.71E-16 |
| RHOA | ENSG00000067560 | up | protein_coding | 4.584212 | 9.36E-16 | 2.71E-16 |
| CALR | ENSG00000179218 | down | protein_coding | -3.49163 | 1.36E-15 | 3.95E-16 |
| CCDC85B | ENSG00000175602 | up | protein_coding | 5.500148 | 1.37E-15 | 3.99E-16 |
| EEF1B2 | ENSG00000114942 | down | protein_coding | -5.60314 | 3.32E-15 | 9.72E-16 |
| RAB2A | ENSG00000104388 | up | protein_coding | 4.528717 | 3.94E-15 | 1.16E-15 |
| CD44 | ENSG00000026508 | up | protein_coding | 5.441254 | 5.37E-15 | 1.59E-15 |
| FADS2 | ENSG00000134824 | up | protein_coding | 5.441254 | 5.37E-15 | 1.59E-15 |
| RABGAP1 | ENSG00000011454 | up | protein_coding | 5.441254 | 5.37E-15 | 1.59E-15 |
| FRMD3 | ENSG00000172159 | up | protein_coding | 5.441254 | 5.37E-15 | 1.59E-15 |
| RPL13 | ENSG00000167526 | up | protein_coding | 5.426147 | 7.53E-15 | 2.25E-15 |
| YLPM1 | ENSG00000119596 | up | protein_coding | 5.410881 | 1.06E-14 | 3.18E-15 |
| PHC2 | ENSG00000134686 | up | protein_coding | 5.410881 | 1.06E-14 | 3.18E-15 |
| SRP9 | ENSG00000143742 | up | protein_coding | 5.395451 | 1.49E-14 | 4.50E-15 |
| MYL12A | ENSG00000101608 | up | protein_coding | 5.395451 | 1.49E-14 | 4.50E-15 |
| TEC | ENSG00000135605 | up | protein_coding | 5.395451 | 1.49E-14 | 4.50E-15 |
| GNB1 | ENSG00000078369 | down | protein_coding | -1.53073 | 1.82E-14 | 5.52E-15 |
| EPB41L2 | ENSG00000079819 | down | protein_coding | -1.3814 | 2.02E-14 | 6.13E-15 |
| SF3B2 | ENSG00000087365 | up | protein_coding | 5.379854 | 2.08E-14 | 6.37E-15 |
| TBL2 | ENSG00000106638 | up | protein_coding | 5.379854 | 2.08E-14 | 6.37E-15 |
| RPS16 | ENSG00000105193 | up | protein_coding | 4.456205 | 2.32E-14 | 7.13E-15 |
| MYO18A | ENSG00000196535 | down | protein_coding | -7.60314 | 4.25E-14 | 1.31E-14 |
| PLEKHM2 | ENSG00000116786 | up | protein_coding | 4.426147 | 4.78E-14 | 1.48E-14 |
| SLTM | ENSG00000137776 | up | protein_coding | 5.332025 | 5.85E-14 | 1.81E-14 |
| NCOA3 | ENSG00000124151 | up | protein_coding | 5.315724 | 8.26E-14 | 2.57E-14 |
| SNX2 | ENSG00000205302 | up | protein_coding | 5.315724 | 8.26E-14 | 2.57E-14 |
| GTF3A | ENSG00000122034 | up | protein_coding | 5.299235 | 1.17E-13 | 3.64E-14 |
| KIAA1217 | ENSG00000120549 | down | protein_coding | -7.53603 | 1.40E-13 | 4.40E-14 |
| TNFAIP2 | ENSG00000185215 | up | protein_coding | 4.379854 | 1.40E-13 | 4.41E-14 |
| TAP1 | ENSG00000168394 | up | protein_coding | 5.282557 | 1.63E-13 | 5.17E-14 |
| TXNDC9 | ENSG00000115514 | up | protein_coding | 5.282557 | 1.63E-13 | 5.17E-14 |
| ARHGAP30 | ENSG00000186517 | up | protein_coding | 5.282557 | 1.63E-13 | 5.17E-14 |
| B3GNT5 | ENSG00000176597 | up | protein_coding | 5.282557 | 1.63E-13 | 5.17E-14 |
| GCC2 | ENSG00000135968 | up | protein_coding | 5.282557 | 1.63E-13 | 5.17E-14 |
| XRCC5 | ENSG00000079246 | up | protein_coding | 5.265683 | 2.30E-13 | 7.35E-14 |
| CLIC1 | ENSG00000213719 | up | protein_coding | 5.248609 | 3.25E-13 | 1.04E-13 |
| ALYREF | ENSG00000183684 | up | protein_coding | 5.248609 | 3.25E-13 | 1.04E-13 |
| PSME1 | ENSG00000092010 | up | protein_coding | 5.248609 | 3.25E-13 | 1.04E-13 |
| HSPA8 | ENSG00000109971 | up | protein_coding | 4.332025 | 4.09E-13 | 1.32E-13 |
| GNG5 | ENSG00000174021 | up | protein_coding | 5.231331 | 4.59E-13 | 1.48E-13 |
| CCDC90B | ENSG00000137500 | down | protein_coding | -7.46564 | 4.61E-13 | 1.50E-13 |
| PSMC6 | ENSG00000100519 | up | protein_coding | 4.315724 | 5.84E-13 | 1.90E-13 |
| SAT1 | ENSG00000130066 | up | protein_coding | 5.213844 | 6.44E-13 | 2.11E-13 |
| LAP3 | ENSG00000002549 | up | protein_coding | 5.213844 | 6.44E-13 | 2.11E-13 |
| CXorf38 | ENSG00000185753 | up | protein_coding | 5.213844 | 6.44E-13 | 2.11E-13 |
| TXNL4A | ENSG00000141759 | up | protein_coding | 5.213844 | 6.44E-13 | 2.11E-13 |
| CXCL8 | ENSG00000169429 | up | protein_coding | 5.213844 | 6.44E-13 | 2.11E-13 |
| GNA13 | ENSG00000120063 | up | protein_coding | 4.299235 | 8.29E-13 | 2.74E-13 |
| PHF14 | ENSG00000106443 | up | protein_coding | 5.196142 | 9.05E-13 | 3.00E-13 |
| RAB5B | ENSG00000111540 | up | protein_coding | 5.196142 | 9.05E-13 | 3.00E-13 |
| KARS | ENSG00000065427 | up | protein_coding | 5.17822 | 1.28E-12 | 4.27E-13 |
| MTATP6P1 | ENSG00000248527 | down | unprocessed_pseudogene | -6.31363 | 1.38E-12 | 4.60E-13 |
| FCGR3A | ENSG00000203747 | up | protein_coding | 5.160073 | 1.81E-12 | 6.09E-13 |
| GUCD1 | ENSG00000138867 | up | protein_coding | 5.160073 | 1.81E-12 | 6.09E-13 |
| TNFAIP3 | ENSG00000118503 | up | protein_coding | 5.160073 | 1.81E-12 | 6.09E-13 |
| CBX3 | ENSG00000122565 | up | protein_coding | 5.160073 | 1.81E-12 | 6.09E-13 |
| SENP7 | ENSG00000138468 | up | protein_coding | 5.141694 | 2.55E-12 | 8.67E-13 |
| EXOC7 | ENSG00000182473 | up | protein_coding | 5.141694 | 2.55E-12 | 8.67E-13 |
| AL450405.1 | ENSG00000230202 | up | processed_pseudogene | 5.141694 | 2.55E-12 | 8.67E-13 |
| RACK1 | ENSG00000204628 | up | protein_coding | 5.141694 | 2.55E-12 | 8.67E-13 |
| TUBGCP2 | ENSG00000130640 | up | protein_coding | 5.123078 | 3.59E-12 | 1.24E-12 |
| RAD21 | ENSG00000164754 | up | protein_coding | 5.123078 | 3.59E-12 | 1.24E-12 |
| CYP1B1 | ENSG00000138061 | up | protein_coding | 5.123078 | 3.59E-12 | 1.24E-12 |
| PPP2R2C | ENSG00000074211 | up | protein_coding | 5.123078 | 3.59E-12 | 1.24E-12 |
| TM9SF3 | ENSG00000077147 | up | protein_coding | 5.123078 | 3.59E-12 | 1.24E-12 |
| CD247 | ENSG00000198821 | up | protein_coding | 5.123078 | 3.59E-12 | 1.24E-12 |
| HNRNPA1 | ENSG00000135486 | up | protein_coding | 5.123078 | 3.59E-12 | 1.24E-12 |
| C11orf58 | ENSG00000110696 | up | protein_coding | 5.104219 | 5.06E-12 | 1.76E-12 |
| NIP7 | ENSG00000132603 | up | protein_coding | 5.104219 | 5.06E-12 | 1.76E-12 |
| TMEM70 | ENSG00000175606 | up | protein_coding | 5.104219 | 5.06E-12 | 1.76E-12 |
| PTPN11 | ENSG00000179295 | up | protein_coding | 4.196142 | 7.04E-12 | 2.47E-12 |
| TTR | ENSG00000118271 | up | protein_coding | 5.085111 | 7.12E-12 | 2.52E-12 |
| KPNB1 | ENSG00000108424 | up | protein_coding | 5.085111 | 7.12E-12 | 2.52E-12 |
| CSDE1 | ENSG00000009307 | up | protein_coding | 5.085111 | 7.12E-12 | 2.52E-12 |
| BIRC3 | ENSG00000023445 | up | protein_coding | 5.085111 | 7.12E-12 | 2.52E-12 |
| DHX29 | ENSG00000067248 | up | protein_coding | 5.085111 | 7.12E-12 | 2.52E-12 |
| CCT5 | ENSG00000150753 | up | protein_coding | 5.085111 | 7.12E-12 | 2.52E-12 |
| EIF2AK2 | ENSG00000055332 | up | protein_coding | 5.065745 | 1.01E-11 | 3.59E-12 |
| UACA | ENSG00000137831 | up | protein_coding | 5.065745 | 1.01E-11 | 3.59E-12 |
| GARS | ENSG00000106105 | up | protein_coding | 5.065745 | 1.01E-11 | 3.59E-12 |
| KMT5B | ENSG00000110066 | up | protein_coding | 5.046116 | 1.43E-11 | 5.13E-12 |
| SFRP5 | ENSG00000120057 | up | protein_coding | 5.046116 | 1.43E-11 | 5.13E-12 |
| C15orf52 | ENSG00000188549 | down | protein_coding | -7.23117 | 1.77E-11 | 6.39E-12 |
| MTDH | ENSG00000147649 | down | protein_coding | -7.23117 | 1.77E-11 | 6.39E-12 |
| IQGAP1 | ENSG00000140575 | up | protein_coding | 5.026217 | 2.02E-11 | 7.33E-12 |
| CAMSAP2 | ENSG00000118200 | up | protein_coding | 5.026217 | 2.02E-11 | 7.33E-12 |
| DST | ENSG00000151914 | up | protein_coding | 5.026217 | 2.02E-11 | 7.33E-12 |
| SP4 | ENSG00000105866 | up | protein_coding | 5.006039 | 2.86E-11 | 1.05E-11 |
| TNKS1BP1 | ENSG00000149115 | up | protein_coding | 5.006039 | 2.86E-11 | 1.05E-11 |
| HLA-DRB1 | ENSG00000196126 | up | protein_coding | 5.006039 | 2.86E-11 | 1.05E-11 |
| NDUFAF8 | ENSG00000224877 | up | protein_coding | 5.006039 | 2.86E-11 | 1.05E-11 |
| EIF4G2 | ENSG00000110321 | up | protein_coding | 3.315724 | 4.91E-11 | 1.81E-11 |
| BCL2A1 | ENSG00000140379 | up | protein_coding | 4.964816 | 5.79E-11 | 2.15E-11 |
| NBN | ENSG00000104320 | up | protein_coding | 4.964816 | 5.79E-11 | 2.15E-11 |
| CCNC | ENSG00000112237 | up | protein_coding | 4.964816 | 5.79E-11 | 2.15E-11 |
| LITAF | ENSG00000189067 | up | protein_coding | 4.964816 | 5.79E-11 | 2.15E-11 |
| PLIN1 | ENSG00000166819 | down | protein_coding | -7.14371 | 6.17E-11 | 2.30E-11 |
| PARP14 | ENSG00000173193 | up | protein_coding | 4.943755 | 8.19E-11 | 3.07E-11 |
| STARD13 | ENSG00000133121 | up | protein_coding | 4.943755 | 8.19E-11 | 3.07E-11 |
| RPAP3 | ENSG00000005175 | up | protein_coding | 4.943755 | 8.19E-11 | 3.07E-11 |
| FMO4 | ENSG00000076258 | up | protein_coding | 4.943755 | 8.19E-11 | 3.07E-11 |
| PPP4R3B | ENSG00000275052 | up | protein_coding | 4.065745 | 8.55E-11 | 3.23E-11 |
| SAMHD1 | ENSG00000101347 | up | protein_coding | 2.036201 | 9.92E-11 | 3.75E-11 |
| PTN | ENSG00000105894 | up | protein_coding | 4.922381 | 1.16E-10 | 4.40E-11 |
| PSME2 | ENSG00000100911 | up | protein_coding | 4.922381 | 1.16E-10 | 4.40E-11 |
| CRIPT | ENSG00000119878 | up | protein_coding | 4.900686 | 1.65E-10 | 6.31E-11 |
| MLLT6 | ENSG00000275023 | up | protein_coding | 4.900686 | 1.65E-10 | 6.31E-11 |
| TRPM3 | ENSG00000083067 | up | protein_coding | 4.900686 | 1.65E-10 | 6.31E-11 |
| AC103691.1 | ENSG00000274383 | up | antisense_RNA | 4.900686 | 1.65E-10 | 6.31E-11 |
| ADGRE5 | ENSG00000123146 | up | protein_coding | 4.87866 | 2.35E-10 | 9.05E-11 |
| LARP4B | ENSG00000107929 | up | protein_coding | 4.87866 | 2.35E-10 | 9.05E-11 |
| MCL1 | ENSG00000143384 | up | protein_coding | 2.570537 | 2.44E-10 | 9.42E-11 |
| CXCL6 | ENSG00000124875 | up | protein_coding | 4.856292 | 3.35E-10 | 1.30E-10 |
| EXT1 | ENSG00000182197 | up | protein_coding | 4.856292 | 3.35E-10 | 1.30E-10 |
| DCAF12 | ENSG00000198876 | up | protein_coding | 4.856292 | 3.35E-10 | 1.30E-10 |
| TGM2 | ENSG00000198959 | up | protein_coding | 4.856292 | 3.35E-10 | 1.30E-10 |
| NUDT4 | ENSG00000173598 | up | protein_coding | 4.833572 | 4.76E-10 | 1.86E-10 |
| HNRNPM | ENSG00000099783 | up | protein_coding | 4.833572 | 4.76E-10 | 1.86E-10 |
| FAM76B | ENSG00000077458 | up | protein_coding | 4.833572 | 4.76E-10 | 1.86E-10 |
| ARID5B | ENSG00000150347 | up | protein_coding | 4.833572 | 4.76E-10 | 1.86E-10 |
| FRMD4B | ENSG00000114541 | up | protein_coding | 4.810488 | 6.77E-10 | 2.68E-10 |
| AHSA1 | ENSG00000100591 | up | protein_coding | 4.810488 | 6.77E-10 | 2.68E-10 |
| MTHFD2 | ENSG00000065911 | up | protein_coding | 4.810488 | 6.77E-10 | 2.68E-10 |
| ARHGEF12 | ENSG00000196914 | up | protein_coding | 4.810488 | 6.77E-10 | 2.68E-10 |
| SAMD9 | ENSG00000205413 | up | protein_coding | 4.810488 | 6.77E-10 | 2.68E-10 |
| CEP57 | ENSG00000166037 | down | protein_coding | -6.95106 | 7.89E-10 | 3.14E-10 |
| SMS | ENSG00000102172 | down | protein_coding | -6.95106 | 7.89E-10 | 3.14E-10 |
| GPR153 | ENSG00000158292 | up | protein_coding | 4.787029 | 9.60E-10 | 3.85E-10 |
| OTUD5 | ENSG00000068308 | up | protein_coding | 4.787029 | 9.60E-10 | 3.85E-10 |
| AC106786.1 | ENSG00000223652 | up | antisense_RNA | 4.787029 | 9.60E-10 | 3.85E-10 |
| DOHH | ENSG00000129932 | up | protein_coding | 4.763182 | 1.37E-09 | 5.53E-10 |
| FAM129B | ENSG00000136830 | up | protein_coding | 4.763182 | 1.37E-09 | 5.53E-10 |
| HNRNPH3 | ENSG00000096746 | up | protein_coding | 4.763182 | 1.37E-09 | 5.53E-10 |
| CLIP2 | ENSG00000106665 | up | protein_coding | 4.763182 | 1.37E-09 | 5.53E-10 |
| STAT1 | ENSG00000115415 | up | protein_coding | 4.738935 | 1.95E-09 | 7.96E-10 |
| PARP9 | ENSG00000138496 | up | protein_coding | 4.738935 | 1.95E-09 | 7.96E-10 |
| RPS2P5 | ENSG00000240342 | up | processed_pseudogene | 4.738935 | 1.95E-09 | 7.96E-10 |
| SIN3B | ENSG00000127511 | up | protein_coding | 4.738935 | 1.95E-09 | 7.96E-10 |
| CCT6A | ENSG00000146731 | up | protein_coding | 4.738935 | 1.95E-09 | 7.96E-10 |
| SH2B1 | ENSG00000178188 | up | protein_coding | 4.714273 | 2.79E-09 | 1.15E-09 |
| PSMD4 | ENSG00000159352 | up | protein_coding | 4.714273 | 2.79E-09 | 1.15E-09 |
| TCP11X3P | ENSG00000251525 | down | transcribed_unprocessed_pseudogene | -6.84415 | 2.89E-09 | 1.19E-09 |
| SNTA1 | ENSG00000101400 | down | protein_coding | -6.84415 | 2.89E-09 | 1.19E-09 |
| EIF5A | ENSG00000132507 | up | protein_coding | 3.856292 | 3.10E-09 | 1.28E-09 |
| MT-ND1 | ENSG00000198888 | up | protein_coding | 1.582117 | 3.38E-09 | 1.40E-09 |
| PACSIN2 | ENSG00000100266 | up | protein_coding | 4.689182 | 3.97E-09 | 1.65E-09 |
| SNX3 | ENSG00000112335 | up | protein_coding | 4.689182 | 3.97E-09 | 1.65E-09 |
| DNAJB1 | ENSG00000132002 | up | protein_coding | 3.833572 | 4.44E-09 | 1.85E-09 |
| USP8 | ENSG00000138592 | up | protein_coding | 4.663647 | 5.67E-09 | 2.38E-09 |
| PIN4 | ENSG00000102309 | up | protein_coding | 4.663647 | 5.67E-09 | 2.38E-09 |
| IFI27 | ENSG00000165949 | up | protein_coding | 4.663647 | 5.67E-09 | 2.38E-09 |
| LRRC75A-AS1 | ENSG00000175061 | up | processed_transcript | 3.810488 | 6.37E-09 | 2.68E-09 |
| RPS19 | ENSG00000105372 | down | protein_coding | -2.20511 | 6.43E-09 | 2.71E-09 |
| PRPF31 | ENSG00000105618 | up | protein_coding | 4.637652 | 8.06E-09 | 3.43E-09 |
| AEBP2 | ENSG00000139154 | up | protein_coding | 4.637652 | 8.06E-09 | 3.43E-09 |
| GRK6 | ENSG00000198055 | up | protein_coding | 4.637652 | 8.06E-09 | 3.43E-09 |
| RPS19BP1 | ENSG00000187051 | up | protein_coding | 4.637652 | 8.06E-09 | 3.43E-09 |
| MRPL41 | ENSG00000182154 | up | protein_coding | 4.637652 | 8.06E-09 | 3.43E-09 |
| AKR1A1 | ENSG00000117448 | up | protein_coding | 4.637652 | 8.06E-09 | 3.43E-09 |
| ARID4B | ENSG00000054267 | up | protein_coding | 4.637652 | 8.06E-09 | 3.43E-09 |
| RPS6 | ENSG00000137154 | up | protein_coding | 3.046116 | 8.88E-09 | 3.81E-09 |
| CEP295 | ENSG00000166004 | down | protein_coding | -6.72867 | 1.08E-08 | 4.63E-09 |
| NDUFS5 | ENSG00000168653 | up | protein_coding | 4.611179 | 1.14E-08 | 4.94E-09 |
| SMARCC2 | ENSG00000139613 | up | protein_coding | 4.611179 | 1.14E-08 | 4.94E-09 |
| RBP1 | ENSG00000114115 | up | protein_coding | 4.611179 | 1.14E-08 | 4.94E-09 |
| RPL10A | ENSG00000198755 | up | protein_coding | 4.611179 | 1.14E-08 | 4.94E-09 |
| CNOT1 | ENSG00000125107 | up | protein_coding | 3.026217 | 1.25E-08 | 5.42E-09 |
| BHLHE40 | ENSG00000134107 | up | protein_coding | 4.584212 | 1.63E-08 | 7.13E-09 |
| STX12 | ENSG00000117758 | up | protein_coding | 4.584212 | 1.63E-08 | 7.13E-09 |
| GNAS | ENSG00000087460 | up | protein_coding | 4.584212 | 1.63E-08 | 7.13E-09 |
| RPS21 | ENSG00000171858 | up | protein_coding | 4.584212 | 1.63E-08 | 7.13E-09 |
| UBA52P5 | ENSG00000243498 | up | processed_pseudogene | 4.584212 | 1.63E-08 | 7.13E-09 |
| KAT6B | ENSG00000156650 | up | protein_coding | 3.738935 | 1.84E-08 | 8.10E-09 |
| HNRNPA1P48 | ENSG00000224578 | up | transcribed_processed_pseudogene | 3.738935 | 1.84E-08 | 8.10E-09 |
| GALNT3 | ENSG00000115339 | up | protein_coding | 4.556732 | 2.31E-08 | 1.03E-08 |
| RUFY4 | ENSG00000188282 | up | protein_coding | 4.556732 | 2.31E-08 | 1.03E-08 |
| RAB11FIP3 | ENSG00000090565 | up | protein_coding | 4.556732 | 2.31E-08 | 1.03E-08 |
| DHX15 | ENSG00000109606 | up | protein_coding | 4.556732 | 2.31E-08 | 1.03E-08 |
| NACA | ENSG00000196531 | up | protein_coding | 4.556732 | 2.31E-08 | 1.03E-08 |
| CLNS1A | ENSG00000074201 | up | protein_coding | 4.556732 | 2.31E-08 | 1.03E-08 |
| CFL1 | ENSG00000172757 | up | protein_coding | 4.556732 | 2.31E-08 | 1.03E-08 |
| GPX3 | ENSG00000211445 | up | protein_coding | 4.556732 | 2.31E-08 | 1.03E-08 |
| IFIT1 | ENSG00000185745 | up | protein_coding | 4.556732 | 2.31E-08 | 1.03E-08 |
| SLC22A3 | ENSG00000146477 | up | protein_coding | 4.556732 | 2.31E-08 | 1.03E-08 |
| SLC22A17 | ENSG00000092096 | up | protein_coding | 4.556732 | 2.31E-08 | 1.03E-08 |
| PTPN2 | ENSG00000175354 | up | protein_coding | 4.556732 | 2.31E-08 | 1.03E-08 |
| WDR13 | ENSG00000101940 | up | protein_coding | 4.556732 | 2.31E-08 | 1.03E-08 |
| COX5B | ENSG00000135940 | up | protein_coding | 4.556732 | 2.31E-08 | 1.03E-08 |
| SLC25A6 | ENSG00000169100 | up | protein_coding | 3.714273 | 2.58E-08 | 1.17E-08 |
| RAPH1 | ENSG00000173166 | up | protein_coding | 4.528717 | 3.26E-08 | 1.49E-08 |
| PLPP3 | ENSG00000162407 | up | protein_coding | 4.528717 | 3.26E-08 | 1.49E-08 |
| RUFY1 | ENSG00000176783 | up | protein_coding | 4.528717 | 3.26E-08 | 1.49E-08 |
| SMG5 | ENSG00000198952 | up | protein_coding | 4.528717 | 3.26E-08 | 1.49E-08 |
| SUDS3 | ENSG00000111707 | up | protein_coding | 4.528717 | 3.26E-08 | 1.49E-08 |
| RPS6KA6 | ENSG00000072133 | up | protein_coding | 4.528717 | 3.26E-08 | 1.49E-08 |
| SIDT1 | ENSG00000072858 | down | protein_coding | -6.60314 | 3.99E-08 | 1.83E-08 |
| SLC25A39 | ENSG00000013306 | down | protein_coding | -6.60314 | 3.99E-08 | 1.83E-08 |
| WDR1 | ENSG00000071127 | up | protein_coding | 4.500148 | 4.63E-08 | 2.15E-08 |
| RBM25 | ENSG00000119707 | up | protein_coding | 4.500148 | 4.63E-08 | 2.15E-08 |
| MPHOSPH8 | ENSG00000196199 | up | protein_coding | 4.500148 | 4.63E-08 | 2.15E-08 |
| MEX3C | ENSG00000176624 | up | protein_coding | 4.500148 | 4.63E-08 | 2.15E-08 |
| PRAME | ENSG00000185686 | up | protein_coding | 4.500148 | 4.63E-08 | 2.15E-08 |
| SAMD1 | ENSG00000141858 | up | protein_coding | 4.500148 | 4.63E-08 | 2.15E-08 |
| COPZ1 | ENSG00000111481 | up | protein_coding | 4.500148 | 4.63E-08 | 2.15E-08 |
| STX6 | ENSG00000135823 | up | protein_coding | 4.500148 | 4.63E-08 | 2.15E-08 |
| PIKFYVE | ENSG00000115020 | up | protein_coding | 3.663647 | 5.23E-08 | 2.45E-08 |
| YBX1 | ENSG00000065978 | up | protein_coding | 2.556732 | 5.60E-08 | 2.62E-08 |
| FAF1 | ENSG00000185104 | up | protein_coding | 2.556732 | 5.60E-08 | 2.62E-08 |
| SLC1A6 | ENSG00000105143 | up | protein_coding | 4.471002 | 6.57E-08 | 3.11E-08 |
| PKP4 | ENSG00000144283 | up | protein_coding | 4.471002 | 6.57E-08 | 3.11E-08 |
| TSR1 | ENSG00000167721 | up | protein_coding | 4.471002 | 6.57E-08 | 3.11E-08 |
| RPL10 | ENSG00000147403 | up | protein_coding | 4.471002 | 6.57E-08 | 3.11E-08 |
| RPS12 | ENSG00000112306 | up | protein_coding | 4.471002 | 6.57E-08 | 3.11E-08 |
| TP63 | ENSG00000073282 | up | protein_coding | 4.471002 | 6.57E-08 | 3.11E-08 |
| NEK7 | ENSG00000151414 | up | protein_coding | 4.471002 | 6.57E-08 | 3.11E-08 |
| SHC1 | ENSG00000160691 | up | protein_coding | 4.471002 | 6.57E-08 | 3.11E-08 |
| LDLR | ENSG00000130164 | up | protein_coding | 4.441254 | 9.39E-08 | 4.50E-08 |
| PPFIBP1 | ENSG00000110841 | up | protein_coding | 4.441254 | 9.39E-08 | 4.50E-08 |
| ZNF585B | ENSG00000245680 | up | protein_coding | 4.441254 | 9.39E-08 | 4.50E-08 |
| GPT2 | ENSG00000166123 | up | protein_coding | 4.441254 | 9.39E-08 | 4.50E-08 |
| RPL23 | ENSG00000125691 | up | protein_coding | 4.441254 | 9.39E-08 | 4.50E-08 |
| DEDD2 | ENSG00000160570 | up | protein_coding | 3.611179 | 1.06E-07 | 5.11E-08 |
| AL078639.1 | ENSG00000281508 | down | antisense_RNA | -5.46564 | 1.32E-07 | 6.36E-08 |
| HLA-C | ENSG00000204525 | down | protein_coding | -5.46564 | 1.32E-07 | 6.36E-08 |
| NAMPT | ENSG00000105835 | up | protein_coding | 4.410881 | 1.34E-07 | 6.51E-08 |
| RPLP0 | ENSG00000089157 | up | protein_coding | 4.410881 | 1.34E-07 | 6.51E-08 |
| RBM23 | ENSG00000100461 | up | protein_coding | 4.410881 | 1.34E-07 | 6.51E-08 |
| SNAP29 | ENSG00000099940 | up | protein_coding | 4.410881 | 1.34E-07 | 6.51E-08 |
| TMEM44-AS1 | ENSG00000231770 | up | antisense_RNA | 4.410881 | 1.34E-07 | 6.51E-08 |
| KLF10 | ENSG00000155090 | up | protein_coding | 4.410881 | 1.34E-07 | 6.51E-08 |
| TMEM18 | ENSG00000151353 | up | protein_coding | 4.410881 | 1.34E-07 | 6.51E-08 |
| CPSF6 | ENSG00000111605 | up | protein_coding | 4.410881 | 1.34E-07 | 6.51E-08 |
| IFIT3 | ENSG00000119917 | up | protein_coding | 3.584212 | 1.50E-07 | 7.39E-08 |
| GNPDA1 | ENSG00000113552 | down | protein_coding | -6.46564 | 1.51E-07 | 7.45E-08 |
| DYNLL2 | ENSG00000264364 | down | protein_coding | -6.46564 | 1.51E-07 | 7.45E-08 |
| EAF1 | ENSG00000144597 | up | protein_coding | 4.379854 | 1.90E-07 | 9.43E-08 |
| SVIL | ENSG00000197321 | up | protein_coding | 4.379854 | 1.90E-07 | 9.43E-08 |
| CCT3 | ENSG00000163468 | up | protein_coding | 4.379854 | 1.90E-07 | 9.43E-08 |
| ERI3 | ENSG00000117419 | up | protein_coding | 4.379854 | 1.90E-07 | 9.43E-08 |
| PSMD10 | ENSG00000101843 | up | protein_coding | 4.379854 | 1.90E-07 | 9.43E-08 |
| MDFIC | ENSG00000135272 | up | protein_coding | 4.379854 | 1.90E-07 | 9.43E-08 |
| HK2 | ENSG00000159399 | up | protein_coding | 4.379854 | 1.90E-07 | 9.43E-08 |
| AHCYL1 | ENSG00000168710 | up | protein_coding | 4.348145 | 2.72E-07 | 1.37E-07 |
| SYNJ2BP | ENSG00000213463 | up | protein_coding | 4.348145 | 2.72E-07 | 1.37E-07 |
| SRSF5 | ENSG00000100650 | up | protein_coding | 4.348145 | 2.72E-07 | 1.37E-07 |
| TUBA1A | ENSG00000167552 | up | protein_coding | 4.348145 | 2.72E-07 | 1.37E-07 |
| DUT | ENSG00000128951 | up | protein_coding | 4.348145 | 2.72E-07 | 1.37E-07 |
| IFI35 | ENSG00000068079 | up | protein_coding | 4.348145 | 2.72E-07 | 1.37E-07 |
| HIVEP2 | ENSG00000010818 | up | protein_coding | 4.348145 | 2.72E-07 | 1.37E-07 |
| HMGN2 | ENSG00000198830 | up | protein_coding | 4.348145 | 2.72E-07 | 1.37E-07 |
| AC133065.2 | ENSG00000262222 | down | antisense_RNA | -4.46564 | 2.92E-07 | 1.48E-07 |
| HNRNPAB | ENSG00000197451 | up | protein_coding | 4.315724 | 3.88E-07 | 1.98E-07 |
| TCF4 | ENSG00000196628 | up | protein_coding | 4.315724 | 3.88E-07 | 1.98E-07 |
| TBC1D12 | ENSG00000108239 | up | protein_coding | 4.315724 | 3.88E-07 | 1.98E-07 |
| SOD1 | ENSG00000142168 | up | protein_coding | 4.315724 | 3.88E-07 | 1.98E-07 |
| IFITM1 | ENSG00000185885 | up | protein_coding | 4.315724 | 3.88E-07 | 1.98E-07 |
| NCBP1 | ENSG00000136937 | up | protein_coding | 4.315724 | 3.88E-07 | 1.98E-07 |
| HIKESHI | ENSG00000149196 | up | protein_coding | 4.315724 | 3.88E-07 | 1.98E-07 |
| RPL4 | ENSG00000174444 | up | protein_coding | 2.421077 | 5.15E-07 | 2.65E-07 |
| RNF213 | ENSG00000173821 | up | protein_coding | 4.282557 | 5.53E-07 | 2.87E-07 |
| NVL | ENSG00000143748 | up | protein_coding | 4.282557 | 5.53E-07 | 2.87E-07 |
| SPG11 | ENSG00000104133 | up | protein_coding | 4.282557 | 5.53E-07 | 2.87E-07 |
| ZUFSP | ENSG00000153975 | up | protein_coding | 4.282557 | 5.53E-07 | 2.87E-07 |
| SMAD5 | ENSG00000113658 | up | protein_coding | 4.282557 | 5.53E-07 | 2.87E-07 |
| SETBP1 | ENSG00000152217 | up | protein_coding | 4.282557 | 5.53E-07 | 2.87E-07 |
| RPS8 | ENSG00000142937 | up | protein_coding | 4.282557 | 5.53E-07 | 2.87E-07 |
| MTFMT | ENSG00000103707 | up | protein_coding | 4.282557 | 5.53E-07 | 2.87E-07 |
| LINC02169 | ENSG00000259517 | up | lincRNA | 4.282557 | 5.53E-07 | 2.87E-07 |
| AIF1L | ENSG00000126878 | up | protein_coding | 4.282557 | 5.53E-07 | 2.87E-07 |
| USP28 | ENSG00000048028 | up | protein_coding | 4.282557 | 5.53E-07 | 2.87E-07 |
| PTMS | ENSG00000159335 | up | protein_coding | 4.282557 | 5.53E-07 | 2.87E-07 |
| NAT10 | ENSG00000135372 | up | protein_coding | 4.282557 | 5.53E-07 | 2.87E-07 |
| RPL3 | ENSG00000100316 | up | protein_coding | 3.471002 | 6.11E-07 | 3.22E-07 |
| KPNA2 | ENSG00000182481 | up | protein_coding | 3.471002 | 6.11E-07 | 3.22E-07 |
| COL3A1 | ENSG00000168542 | up | protein_coding | 4.248609 | 7.83E-07 | 4.17E-07 |
| SEL1L | ENSG00000071537 | up | protein_coding | 4.248609 | 7.83E-07 | 4.17E-07 |
| LDHA | ENSG00000134333 | up | protein_coding | 4.248609 | 7.83E-07 | 4.17E-07 |
| CCDC88C | ENSG00000015133 | up | protein_coding | 4.248609 | 7.83E-07 | 4.17E-07 |
| EDEM2 | ENSG00000088298 | up | protein_coding | 4.248609 | 7.83E-07 | 4.17E-07 |
| ADAMTS6 | ENSG00000049192 | up | protein_coding | 4.248609 | 7.83E-07 | 4.17E-07 |
| TSC22D4 | ENSG00000166925 | up | protein_coding | 4.248609 | 7.83E-07 | 4.17E-07 |
| RAB3IP | ENSG00000127328 | up | protein_coding | 4.248609 | 7.83E-07 | 4.17E-07 |
| ATP5C1 | ENSG00000165629 | up | protein_coding | 4.248609 | 7.83E-07 | 4.17E-07 |
| PDZRN3 | ENSG00000121440 | up | protein_coding | 4.248609 | 7.83E-07 | 4.17E-07 |
| COX7C | ENSG00000127184 | up | protein_coding | 3.441254 | 8.64E-07 | 4.65E-07 |
| MAPK14 | ENSG00000112062 | up | protein_coding | 3.441254 | 8.64E-07 | 4.65E-07 |
| CLIP4 | ENSG00000115295 | up | protein_coding | 4.213844 | 1.11E-06 | 6.06E-07 |
| PLSCR1 | ENSG00000188313 | up | protein_coding | 4.213844 | 1.11E-06 | 6.06E-07 |
| WDR7 | ENSG00000091157 | up | protein_coding | 4.213844 | 1.11E-06 | 6.06E-07 |
| MIR100HG | ENSG00000255248 | up | processed_transcript | 4.213844 | 1.11E-06 | 6.06E-07 |
| HSBP1 | ENSG00000230989 | up | protein_coding | 4.213844 | 1.11E-06 | 6.06E-07 |
| MYL6 | ENSG00000092841 | up | protein_coding | 4.213844 | 1.11E-06 | 6.06E-07 |
| PCGF3 | ENSG00000185619 | up | protein_coding | 4.213844 | 1.11E-06 | 6.06E-07 |
| DSP | ENSG00000096696 | up | protein_coding | 4.213844 | 1.11E-06 | 6.06E-07 |
| CRMP1 | ENSG00000072832 | up | protein_coding | 4.213844 | 1.11E-06 | 6.06E-07 |
| CEP131 | ENSG00000141577 | up | protein_coding | 4.213844 | 1.11E-06 | 6.06E-07 |
| BEX4 | ENSG00000102409 | up | protein_coding | 4.213844 | 1.11E-06 | 6.06E-07 |
| CXCL10 | ENSG00000169245 | up | protein_coding | 4.213844 | 1.11E-06 | 6.06E-07 |
| OAZ2 | ENSG00000180304 | up | protein_coding | 3.410881 | 1.22E-06 | 6.71E-07 |
| C6orf89 | ENSG00000198663 | up | protein_coding | 3.410881 | 1.22E-06 | 6.71E-07 |
| MT-ND4 | ENSG00000198886 | down | protein_coding | -2.92132 | 1.56E-06 | 8.59E-07 |
| STEAP1B | ENSG00000105889 | up | protein_coding | 4.17822 | 1.58E-06 | 8.80E-07 |
| 8-Sep | ENSG00000164402 | up | protein_coding | 4.17822 | 1.58E-06 | 8.80E-07 |
| ISG15 | ENSG00000187608 | up | protein_coding | 4.17822 | 1.58E-06 | 8.80E-07 |
| ERH | ENSG00000100632 | up | protein_coding | 4.17822 | 1.58E-06 | 8.80E-07 |
| ZFYVE21 | ENSG00000100711 | up | protein_coding | 4.17822 | 1.58E-06 | 8.80E-07 |
| FNDC3B | ENSG00000075420 | up | protein_coding | 4.17822 | 1.58E-06 | 8.80E-07 |
| UBTF | ENSG00000108312 | up | protein_coding | 4.17822 | 1.58E-06 | 8.80E-07 |
| SERINC1 | ENSG00000111897 | up | protein_coding | 4.17822 | 1.58E-06 | 8.80E-07 |
| PRDX2 | ENSG00000167815 | up | protein_coding | 3.379854 | 1.73E-06 | 9.68E-07 |
| TMSB4X | ENSG00000205542 | up | protein_coding | 3.379854 | 1.73E-06 | 9.68E-07 |
| GNAI2 | ENSG00000114353 | down | protein_coding | -1.33012 | 1.74E-06 | 9.75E-07 |
| TGFB1 | ENSG00000105329 | up | protein_coding | 4.141694 | 2.26E-06 | 1.28E-06 |
| ZC3HAV1 | ENSG00000105939 | up | protein_coding | 4.141694 | 2.26E-06 | 1.28E-06 |
| ETFA | ENSG00000140374 | up | protein_coding | 4.141694 | 2.26E-06 | 1.28E-06 |
| IPO5 | ENSG00000065150 | up | protein_coding | 4.141694 | 2.26E-06 | 1.28E-06 |
| LMF2 | ENSG00000100258 | up | protein_coding | 4.141694 | 2.26E-06 | 1.28E-06 |
| APOL6 | ENSG00000221963 | up | protein_coding | 4.141694 | 2.26E-06 | 1.28E-06 |
| LYN | ENSG00000254087 | up | protein_coding | 4.141694 | 2.26E-06 | 1.28E-06 |
| WWTR1 | ENSG00000018408 | up | protein_coding | 4.141694 | 2.26E-06 | 1.28E-06 |
| PDCD4 | ENSG00000150593 | up | protein_coding | 4.141694 | 2.26E-06 | 1.28E-06 |
| ZNF436 | ENSG00000125945 | up | protein_coding | 4.141694 | 2.26E-06 | 1.28E-06 |
| FO704657.1 | ENSG00000272004 | up | antisense_RNA | 4.104219 | 3.23E-06 | 1.86E-06 |
| UBA5 | ENSG00000081307 | up | protein_coding | 4.104219 | 3.23E-06 | 1.86E-06 |
| SLC25A46 | ENSG00000164209 | up | protein_coding | 4.104219 | 3.23E-06 | 1.86E-06 |
| PGAP1 | ENSG00000197121 | up | protein_coding | 4.104219 | 3.23E-06 | 1.86E-06 |
| CHD8 | ENSG00000100888 | up | protein_coding | 4.104219 | 3.23E-06 | 1.86E-06 |
| SEC22B | ENSG00000265808 | up | protein_coding | 4.104219 | 3.23E-06 | 1.86E-06 |
| RPS6KA4 | ENSG00000162302 | up | protein_coding | 4.104219 | 3.23E-06 | 1.86E-06 |
| TCERG1 | ENSG00000113649 | up | protein_coding | 4.104219 | 3.23E-06 | 1.86E-06 |
| RPL38 | ENSG00000172809 | up | protein_coding | 4.104219 | 3.23E-06 | 1.86E-06 |
| GBP1 | ENSG00000117228 | up | protein_coding | 4.104219 | 3.23E-06 | 1.86E-06 |
| PPP1R10 | ENSG00000204569 | up | protein_coding | 4.104219 | 3.23E-06 | 1.86E-06 |
| FAM120AOS | ENSG00000188938 | up | protein_coding | 4.104219 | 3.23E-06 | 1.86E-06 |
| HYOU1 | ENSG00000149428 | up | protein_coding | 4.104219 | 3.23E-06 | 1.86E-06 |
| SOGA1 | ENSG00000149639 | up | protein_coding | 4.104219 | 3.23E-06 | 1.86E-06 |
| NPM1P27 | ENSG00000249353 | up | processed_pseudogene | 3.315724 | 3.46E-06 | 2.01E-06 |
| BNIP2 | ENSG00000140299 | up | protein_coding | 4.065745 | 4.61E-06 | 2.70E-06 |
| SBNO1 | ENSG00000139697 | up | protein_coding | 4.065745 | 4.61E-06 | 2.70E-06 |
| CD163 | ENSG00000177575 | up | protein_coding | 4.065745 | 4.61E-06 | 2.70E-06 |
| SIRT2 | ENSG00000068903 | up | protein_coding | 4.065745 | 4.61E-06 | 2.70E-06 |
| AKAP12 | ENSG00000131016 | up | protein_coding | 4.065745 | 4.61E-06 | 2.70E-06 |
| PARK7 | ENSG00000116288 | up | protein_coding | 4.065745 | 4.61E-06 | 2.70E-06 |
| SLA | ENSG00000155926 | up | protein_coding | 4.065745 | 4.61E-06 | 2.70E-06 |
| SP100 | ENSG00000067066 | up | protein_coding | 4.065745 | 4.61E-06 | 2.70E-06 |
| DONSON | ENSG00000159147 | up | protein_coding | 4.065745 | 4.61E-06 | 2.70E-06 |
| SBF1 | ENSG00000100241 | up | protein_coding | 4.026217 | 6.60E-06 | 3.94E-06 |
| DTX3L | ENSG00000163840 | up | protein_coding | 4.026217 | 6.60E-06 | 3.94E-06 |
| NFIA | ENSG00000162599 | up | protein_coding | 4.026217 | 6.60E-06 | 3.94E-06 |
| AMD1 | ENSG00000123505 | up | protein_coding | 4.026217 | 6.60E-06 | 3.94E-06 |
| SBNO2 | ENSG00000064932 | up | protein_coding | 4.026217 | 6.60E-06 | 3.94E-06 |
| AL136298.3 | ENSG00000259086 | up | processed_pseudogene | 4.026217 | 6.60E-06 | 3.94E-06 |
| GIMAP7 | ENSG00000179144 | up | protein_coding | 4.026217 | 6.60E-06 | 3.94E-06 |
| PPP1R11 | ENSG00000204619 | up | protein_coding | 4.026217 | 6.60E-06 | 3.94E-06 |
| SNX9 | ENSG00000130340 | up | protein_coding | 3.985575 | 9.48E-06 | 5.73E-06 |
| GLRX | ENSG00000173221 | up | protein_coding | 3.985575 | 9.48E-06 | 5.73E-06 |
| ANXA2 | ENSG00000182718 | up | protein_coding | 3.985575 | 9.48E-06 | 5.73E-06 |
| RAB8A | ENSG00000167461 | up | protein_coding | 3.985575 | 9.48E-06 | 5.73E-06 |
| TAB2 | ENSG00000055208 | up | protein_coding | 3.985575 | 9.48E-06 | 5.73E-06 |
| MAZ | ENSG00000103495 | up | protein_coding | 3.985575 | 9.48E-06 | 5.73E-06 |
| ACSL1 | ENSG00000151726 | up | protein_coding | 3.985575 | 9.48E-06 | 5.73E-06 |
| RPS6KC1 | ENSG00000136643 | up | protein_coding | 3.985575 | 9.48E-06 | 5.73E-06 |
| UBL3 | ENSG00000122042 | up | protein_coding | 3.985575 | 9.48E-06 | 5.73E-06 |
| BLOC1S3 | ENSG00000189114 | up | protein_coding | 3.985575 | 9.48E-06 | 5.73E-06 |
| LARP7 | ENSG00000174720 | up | protein_coding | 3.985575 | 9.48E-06 | 5.73E-06 |
| TMSB10 | ENSG00000034510 | up | protein_coding | 1.964816 | 1.37E-05 | 8.32E-06 |
| SLU7 | ENSG00000164609 | up | protein_coding | 3.943755 | 1.36E-05 | 8.35E-06 |
| SMARCC1 | ENSG00000173473 | up | protein_coding | 3.943755 | 1.36E-05 | 8.35E-06 |
| TMEM50A | ENSG00000183726 | up | protein_coding | 3.943755 | 1.36E-05 | 8.35E-06 |
| CMPK1 | ENSG00000162368 | up | protein_coding | 3.943755 | 1.36E-05 | 8.35E-06 |
| C1orf52 | ENSG00000162642 | up | protein_coding | 3.943755 | 1.36E-05 | 8.35E-06 |
| SFXN3 | ENSG00000107819 | up | protein_coding | 3.943755 | 1.36E-05 | 8.35E-06 |
| FAM126A | ENSG00000122591 | up | protein_coding | 3.943755 | 1.36E-05 | 8.35E-06 |
| FUNDC2 | ENSG00000165775 | up | protein_coding | 3.943755 | 1.36E-05 | 8.35E-06 |
| MCM3AP | ENSG00000160294 | up | protein_coding | 3.943755 | 1.36E-05 | 8.35E-06 |
| COL1A2 | ENSG00000164692 | down | protein_coding | -1.83885 | 1.40E-05 | 8.66E-06 |
| ABHD12 | ENSG00000100997 | up | protein_coding | 3.17822 | 1.40E-05 | 8.68E-06 |
| PAWR | ENSG00000177425 | up | protein_coding | 3.17822 | 1.40E-05 | 8.68E-06 |
| G0S2 | ENSG00000123689 | up | protein_coding | 3.17822 | 1.40E-05 | 8.68E-06 |
| ARL6IP5 | ENSG00000144746 | up | protein_coding | 3.900686 | 1.95E-05 | 1.22E-05 |
| ATPIF1 | ENSG00000130770 | up | protein_coding | 3.900686 | 1.95E-05 | 1.22E-05 |
| POLR2E | ENSG00000099817 | up | protein_coding | 3.900686 | 1.95E-05 | 1.22E-05 |
| TLK2P1 | ENSG00000226049 | up | processed_pseudogene | 3.900686 | 1.95E-05 | 1.22E-05 |
| S100A9 | ENSG00000163220 | up | protein_coding | 3.900686 | 1.95E-05 | 1.22E-05 |
| HDAC9 | ENSG00000048052 | up | protein_coding | 3.900686 | 1.95E-05 | 1.22E-05 |
| DHX37 | ENSG00000150990 | up | protein_coding | 3.900686 | 1.95E-05 | 1.22E-05 |
| ABLIM1 | ENSG00000099204 | up | protein_coding | 2.153972 | 1.96E-05 | 1.23E-05 |
| RN7SL5P | ENSG00000265735 | up | misc_RNA | 1.11368 | 2.07E-05 | 1.30E-05 |
| USP39 | ENSG00000168883 | up | protein_coding | 3.856292 | 2.79E-05 | 1.77E-05 |
| UNC45A | ENSG00000140553 | up | protein_coding | 3.856292 | 2.79E-05 | 1.77E-05 |
| PBRM1 | ENSG00000163939 | up | protein_coding | 3.856292 | 2.79E-05 | 1.77E-05 |
| AHNAK | ENSG00000124942 | up | protein_coding | 3.856292 | 2.79E-05 | 1.77E-05 |
| CCDC34 | ENSG00000109881 | up | protein_coding | 3.856292 | 2.79E-05 | 1.77E-05 |
| KMT2D | ENSG00000167548 | up | protein_coding | 3.856292 | 2.79E-05 | 1.77E-05 |
| ERICH1 | ENSG00000104714 | up | protein_coding | 3.856292 | 2.79E-05 | 1.77E-05 |
| ZNF880 | ENSG00000221923 | up | protein_coding | 3.856292 | 2.79E-05 | 1.77E-05 |
| MTMR4 | ENSG00000108389 | up | protein_coding | 3.856292 | 2.79E-05 | 1.77E-05 |
| PHACTR2 | ENSG00000112419 | up | protein_coding | 3.856292 | 2.79E-05 | 1.77E-05 |
| TSPAN17 | ENSG00000048140 | up | protein_coding | 3.856292 | 2.79E-05 | 1.77E-05 |
| CPED1 | ENSG00000106034 | up | protein_coding | 3.856292 | 2.79E-05 | 1.77E-05 |
| EPB41L4A-AS1 | ENSG00000224032 | up | lincRNA | 3.856292 | 2.79E-05 | 1.77E-05 |
| DTX1 | ENSG00000135144 | up | protein_coding | 3.856292 | 2.79E-05 | 1.77E-05 |
| NAA25 | ENSG00000111300 | up | protein_coding | 3.856292 | 2.79E-05 | 1.77E-05 |
| TMEM173 | ENSG00000184584 | up | protein_coding | 3.856292 | 2.79E-05 | 1.77E-05 |
| RUFY2 | ENSG00000204130 | up | protein_coding | 3.856292 | 2.79E-05 | 1.77E-05 |
| NUP153 | ENSG00000124789 | up | protein_coding | 3.065745 | 4.00E-05 | 2.58E-05 |
| SMARCA5 | ENSG00000153147 | up | protein_coding | 3.065745 | 4.00E-05 | 2.58E-05 |
| HNRNPA3 | ENSG00000170144 | up | protein_coding | 3.065745 | 4.00E-05 | 2.58E-05 |
| HIRA | ENSG00000100084 | up | protein_coding | 3.810488 | 3.98E-05 | 2.59E-05 |
| GADD45B | ENSG00000099860 | up | protein_coding | 3.810488 | 3.98E-05 | 2.59E-05 |
| SGTA | ENSG00000104969 | up | protein_coding | 3.810488 | 3.98E-05 | 2.59E-05 |
| PPIG | ENSG00000138398 | up | protein_coding | 3.810488 | 3.98E-05 | 2.59E-05 |
| ZNF143 | ENSG00000166478 | up | protein_coding | 3.810488 | 3.98E-05 | 2.59E-05 |
| COTL1 | ENSG00000103187 | up | protein_coding | 3.810488 | 3.98E-05 | 2.59E-05 |
| CACYBP | ENSG00000116161 | up | protein_coding | 3.810488 | 3.98E-05 | 2.59E-05 |
| HSPA1A | ENSG00000204389 | up | protein_coding | 3.810488 | 3.98E-05 | 2.59E-05 |
| EIF5B | ENSG00000158417 | up | protein_coding | 1.358792 | 4.81E-05 | 3.15E-05 |
| CTDSP2 | ENSG00000175215 | up | protein_coding | 3.026217 | 5.66E-05 | 3.71E-05 |
| MAPKAP1 | ENSG00000119487 | up | protein_coding | 3.763182 | 5.68E-05 | 3.77E-05 |
| COQ6 | ENSG00000119723 | up | protein_coding | 3.763182 | 5.68E-05 | 3.77E-05 |
| LETM1 | ENSG00000168924 | up | protein_coding | 3.763182 | 5.68E-05 | 3.77E-05 |
| FKBP8 | ENSG00000105701 | up | protein_coding | 3.763182 | 5.68E-05 | 3.77E-05 |
| ADNP | ENSG00000101126 | up | protein_coding | 3.763182 | 5.68E-05 | 3.77E-05 |
| GNAI3 | ENSG00000065135 | up | protein_coding | 3.763182 | 5.68E-05 | 3.77E-05 |
| MOB1A | ENSG00000114978 | up | protein_coding | 3.763182 | 5.68E-05 | 3.77E-05 |
| UBXN7 | ENSG00000163960 | up | protein_coding | 3.763182 | 5.68E-05 | 3.77E-05 |
| AC072061.1 | ENSG00000259826 | up | antisense_RNA | 3.763182 | 5.68E-05 | 3.77E-05 |
| PPIA | ENSG00000196262 | up | protein_coding | 3.763182 | 5.68E-05 | 3.77E-05 |
| IFI44 | ENSG00000137965 | up | protein_coding | 3.763182 | 5.68E-05 | 3.77E-05 |
| SOBP | ENSG00000112320 | up | protein_coding | 3.763182 | 5.68E-05 | 3.77E-05 |
| ARL4D | ENSG00000175906 | up | protein_coding | 3.763182 | 5.68E-05 | 3.77E-05 |
| RPS7P1 | ENSG00000263266 | up | processed_pseudogene | 3.763182 | 5.68E-05 | 3.77E-05 |
| GFOD2 | ENSG00000141098 | up | protein_coding | 3.763182 | 5.68E-05 | 3.77E-05 |
| ME2 | ENSG00000082212 | up | protein_coding | 3.763182 | 5.68E-05 | 3.77E-05 |
| CYP26B1 | ENSG00000003137 | up | protein_coding | 3.763182 | 5.68E-05 | 3.77E-05 |
| CHMP4B | ENSG00000101421 | up | protein_coding | 3.763182 | 5.68E-05 | 3.77E-05 |
| SERPINB9 | ENSG00000170542 | up | protein_coding | 3.763182 | 5.68E-05 | 3.77E-05 |
| RPL32 | ENSG00000144713 | up | protein_coding | 2.985575 | 7.92E-05 | 5.32E-05 |
| HNRNPF | ENSG00000169813 | up | protein_coding | 2.985575 | 7.92E-05 | 5.32E-05 |
| CDO1 | ENSG00000129596 | up | protein_coding | 3.714273 | 8.14E-05 | 5.50E-05 |
| ADRM1 | ENSG00000130706 | up | protein_coding | 3.714273 | 8.14E-05 | 5.50E-05 |
| SIRT5 | ENSG00000124523 | up | protein_coding | 3.714273 | 8.14E-05 | 5.50E-05 |
| RPS27A | ENSG00000143947 | up | protein_coding | 3.714273 | 8.14E-05 | 5.50E-05 |
| AC019171.1 | ENSG00000269210 | up | antisense_RNA | 3.714273 | 8.14E-05 | 5.50E-05 |
| KRCC1 | ENSG00000172086 | up | protein_coding | 3.714273 | 8.14E-05 | 5.50E-05 |
| PRRC2C | ENSG00000117523 | down | protein_coding | -3.3661 | 8.95E-05 | 6.08E-05 |
| MRFAP1 | ENSG00000179010 | up | protein_coding | 2.943755 | 0.000112287 | 7.64E-05 |
| JMJD4 | ENSG00000081692 | up | protein_coding | 3.663647 | 0.000116765 | 8.03E-05 |
| GSTM3 | ENSG00000134202 | up | protein_coding | 3.663647 | 0.000116765 | 8.03E-05 |
| THNSL2 | ENSG00000144115 | up | protein_coding | 3.663647 | 0.000116765 | 8.03E-05 |
| UBAP2L | ENSG00000143569 | up | protein_coding | 3.663647 | 0.000116765 | 8.03E-05 |
| EIF3J | ENSG00000104131 | up | protein_coding | 3.663647 | 0.000116765 | 8.03E-05 |
| GSDMD | ENSG00000104518 | up | protein_coding | 3.663647 | 0.000116765 | 8.03E-05 |
| SUCO | ENSG00000094975 | up | protein_coding | 3.663647 | 0.000116765 | 8.03E-05 |
| GBP4 | ENSG00000162654 | up | protein_coding | 3.663647 | 0.000116765 | 8.03E-05 |
| AFF1 | ENSG00000172493 | up | protein_coding | 3.663647 | 0.000116765 | 8.03E-05 |
| RN7SL445P | ENSG00000240577 | up | misc_RNA | 3.663647 | 0.000116765 | 8.03E-05 |
| PURB | ENSG00000146676 | up | protein_coding | 3.663647 | 0.000116765 | 8.03E-05 |
| NAMPTP1 | ENSG00000229644 | up | processed_pseudogene | 3.663647 | 0.000116765 | 8.03E-05 |
| ABCF1 | ENSG00000204574 | up | protein_coding | 3.663647 | 0.000116765 | 8.03E-05 |
| PITPNB | ENSG00000180957 | up | protein_coding | 3.663647 | 0.000116765 | 8.03E-05 |
| RPS2P46 | ENSG00000189343 | up | processed_pseudogene | 3.663647 | 0.000116765 | 8.03E-05 |
| VCPIP1 | ENSG00000175073 | up | protein_coding | 3.663647 | 0.000116765 | 8.03E-05 |
| DUSP16 | ENSG00000111266 | up | protein_coding | 3.663647 | 0.000116765 | 8.03E-05 |
| BECN1 | ENSG00000126581 | up | protein_coding | 2.900686 | 0.000157185 | 0.000109414 |
| MARCKS | ENSG00000277443 | up | protein_coding | 2.900686 | 0.000157185 | 0.000109414 |
| ZFHX3 | ENSG00000140836 | up | protein_coding | 1.763182 | 0.000161182 | 0.000112477 |
| RPS2 | ENSG00000140988 | up | protein_coding | 1.763182 | 0.000161182 | 0.000112477 |
| KANSL1L | ENSG00000144445 | up | protein_coding | 3.611179 | 0.000166159 | 0.00011725 |
| ARL1 | ENSG00000120805 | up | protein_coding | 3.611179 | 0.000166159 | 0.00011725 |
| GDI1 | ENSG00000203879 | up | protein_coding | 3.611179 | 0.000166159 | 0.00011725 |
| ARHGEF6 | ENSG00000129675 | up | protein_coding | 3.611179 | 0.000166159 | 0.00011725 |
| GIT2 | ENSG00000139436 | up | protein_coding | 3.611179 | 0.000166159 | 0.00011725 |
| SF3B1 | ENSG00000115524 | up | protein_coding | 3.611179 | 0.000166159 | 0.00011725 |
| SERPING1 | ENSG00000149131 | up | protein_coding | 3.611179 | 0.000166159 | 0.00011725 |
| JOSD1 | ENSG00000100221 | up | protein_coding | 3.611179 | 0.000166159 | 0.00011725 |
| ATP5L | ENSG00000167283 | up | protein_coding | 3.611179 | 0.000166159 | 0.00011725 |
| CAVIN1 | ENSG00000177469 | up | protein_coding | 3.611179 | 0.000166159 | 0.00011725 |
| ZCCHC6 | ENSG00000083223 | up | protein_coding | 3.611179 | 0.000166159 | 0.00011725 |
| DDX58 | ENSG00000107201 | up | protein_coding | 3.611179 | 0.000166159 | 0.00011725 |
| ACTR10 | ENSG00000131966 | up | protein_coding | 3.611179 | 0.000166159 | 0.00011725 |
| DES | ENSG00000175084 | up | protein_coding | 3.611179 | 0.000166159 | 0.00011725 |
| PSAT1 | ENSG00000135069 | up | protein_coding | 3.611179 | 0.000166159 | 0.00011725 |
| CAPRIN1 | ENSG00000135387 | up | protein_coding | 3.611179 | 0.000166159 | 0.00011725 |
| PQLC1 | ENSG00000122490 | up | protein_coding | 2.856292 | 0.000219676 | 0.000156639 |
| AC091980.1 | ENSG00000213393 | up | processed_pseudogene | 3.556732 | 0.0002375 | 0.000171207 |
| PTPRM | ENSG00000173482 | up | protein_coding | 3.556732 | 0.0002375 | 0.000171207 |
| GOLGA3 | ENSG00000090615 | up | protein_coding | 3.556732 | 0.0002375 | 0.000171207 |
| SUPT6H | ENSG00000109111 | up | protein_coding | 3.556732 | 0.0002375 | 0.000171207 |
| TYROBP | ENSG00000011600 | up | protein_coding | 3.556732 | 0.0002375 | 0.000171207 |
| PHRF1 | ENSG00000070047 | up | protein_coding | 3.556732 | 0.0002375 | 0.000171207 |
| PTPN1 | ENSG00000196396 | up | protein_coding | 3.556732 | 0.0002375 | 0.000171207 |
| RPL30 | ENSG00000156482 | up | protein_coding | 3.556732 | 0.0002375 | 0.000171207 |
| BAG5 | ENSG00000166170 | up | protein_coding | 3.556732 | 0.0002375 | 0.000171207 |
| SH3PXD2A | ENSG00000107957 | up | protein_coding | 3.556732 | 0.0002375 | 0.000171207 |
| ECHDC1 | ENSG00000093144 | up | protein_coding | 3.556732 | 0.0002375 | 0.000171207 |
| EID2 | ENSG00000176396 | up | protein_coding | 3.556732 | 0.0002375 | 0.000171207 |
| PDCD6 | ENSG00000249915 | up | protein_coding | 3.556732 | 0.0002375 | 0.000171207 |
| HMGCS1 | ENSG00000112972 | up | protein_coding | 3.556732 | 0.0002375 | 0.000171207 |
| CCDC69 | ENSG00000198624 | up | protein_coding | 3.556732 | 0.0002375 | 0.000171207 |
| PDXK | ENSG00000160209 | up | protein_coding | 3.556732 | 0.0002375 | 0.000171207 |
| NUDT9 | ENSG00000170502 | up | protein_coding | 3.556732 | 0.0002375 | 0.000171207 |
| PKM | ENSG00000067225 | down | protein_coding | -2.55875 | 0.000244809 | 0.000178391 |
| TP53BP2 | ENSG00000143514 | up | protein_coding | 2.810488 | 0.000306687 | 0.000224015 |
| ARHGDIB | ENSG00000111348 | up | protein_coding | 2.810488 | 0.000306687 | 0.000224015 |
| ADAR | ENSG00000160710 | up | protein_coding | 2.810488 | 0.000306687 | 0.000224015 |
| MPP1 | ENSG00000130830 | up | protein_coding | 3.500148 | 0.000339676 | 0.000250031 |
| RMI1 | ENSG00000178966 | up | protein_coding | 3.500148 | 0.000339676 | 0.000250031 |
| MAPKAPK5 | ENSG00000089022 | up | protein_coding | 3.500148 | 0.000339676 | 0.000250031 |
| RPH3A | ENSG00000089169 | up | protein_coding | 3.500148 | 0.000339676 | 0.000250031 |
| TMED8 | ENSG00000100580 | up | protein_coding | 3.500148 | 0.000339676 | 0.000250031 |
| CLINT1 | ENSG00000113282 | up | protein_coding | 3.500148 | 0.000339676 | 0.000250031 |
| MAP1LC3B | ENSG00000140941 | up | protein_coding | 3.500148 | 0.000339676 | 0.000250031 |
| DIXDC1 | ENSG00000150764 | up | protein_coding | 3.500148 | 0.000339676 | 0.000250031 |
| METAP2 | ENSG00000111142 | up | protein_coding | 3.500148 | 0.000339676 | 0.000250031 |
| ZYX | ENSG00000159840 | up | protein_coding | 3.500148 | 0.000339676 | 0.000250031 |
| ZNF660 | ENSG00000144792 | up | protein_coding | 3.441254 | 0.000489746 | 0.00036518 |
| ZAP70 | ENSG00000115085 | up | protein_coding | 3.441254 | 0.000489746 | 0.00036518 |
| SRSF6 | ENSG00000124193 | up | protein_coding | 3.441254 | 0.000489746 | 0.00036518 |
| BMP2K | ENSG00000138756 | up | protein_coding | 3.441254 | 0.000489746 | 0.00036518 |
| EHBP1L1 | ENSG00000173442 | up | protein_coding | 3.441254 | 0.000489746 | 0.00036518 |
| FRA10AC1 | ENSG00000148690 | up | protein_coding | 3.441254 | 0.000489746 | 0.00036518 |
| FAM102B | ENSG00000162636 | up | protein_coding | 3.441254 | 0.000489746 | 0.00036518 |
| TCEAL3 | ENSG00000196507 | up | protein_coding | 3.441254 | 0.000489746 | 0.00036518 |
| GBP3 | ENSG00000117226 | up | protein_coding | 3.441254 | 0.000489746 | 0.00036518 |
| INHBA | ENSG00000122641 | up | protein_coding | 3.441254 | 0.000489746 | 0.00036518 |
| C10orf10 | ENSG00000165507 | up | protein_coding | 3.441254 | 0.000489746 | 0.00036518 |
| DHX8 | ENSG00000067596 | up | protein_coding | 3.441254 | 0.000489746 | 0.00036518 |
| CCSER1 | ENSG00000184305 | down | protein_coding | -2.33635 | 0.000500185 | 0.000375791 |
| S100A10 | ENSG00000197747 | up | protein_coding | 2.714273 | 0.000606687 | 0.000456598 |
| 6-Mar | ENSG00000145495 | up | protein_coding | 2.714273 | 0.000606687 | 0.000456598 |
| FKBP3 | ENSG00000100442 | up | protein_coding | 3.379854 | 0.000704234 | 0.00053338 |
| KPNA4 | ENSG00000186432 | up | protein_coding | 3.379854 | 0.000704234 | 0.00053338 |
| NR3C1 | ENSG00000113580 | up | protein_coding | 3.379854 | 0.000704234 | 0.00053338 |
| BIN3 | ENSG00000147439 | up | protein_coding | 3.379854 | 0.000704234 | 0.00053338 |
| LCP1 | ENSG00000136167 | up | protein_coding | 3.379854 | 0.000704234 | 0.00053338 |
| HNRNPA1P10 | ENSG00000214223 | up | processed_pseudogene | 3.379854 | 0.000704234 | 0.00053338 |
| VPS37A | ENSG00000155975 | up | protein_coding | 3.379854 | 0.000704234 | 0.00053338 |
| RNMT | ENSG00000101654 | up | protein_coding | 3.379854 | 0.000704234 | 0.00053338 |
| CDC42BPB | ENSG00000198752 | up | protein_coding | 3.379854 | 0.000704234 | 0.00053338 |
| NET1 | ENSG00000173848 | up | protein_coding | 2.663647 | 0.000852659 | 0.000650616 |
| POFUT1 | ENSG00000101346 | up | protein_coding | 2.663647 | 0.000852659 | 0.000650616 |
| HTATSF1 | ENSG00000102241 | down | protein_coding | -1.4275 | 0.001000873 | 0.000765015 |
| FAM149A | ENSG00000109794 | down | protein_coding | -2.72867 | 0.001015738 | 0.000777261 |
| PPP2R5E | ENSG00000154001 | up | protein_coding | 3.315724 | 0.001009444 | 0.000779027 |
| VMO1 | ENSG00000182853 | up | protein_coding | 3.315724 | 0.001009444 | 0.000779027 |
| RBMX | ENSG00000147274 | up | protein_coding | 3.315724 | 0.001009444 | 0.000779027 |
| RPGR | ENSG00000156313 | up | protein_coding | 3.315724 | 0.001009444 | 0.000779027 |
| CPPED1 | ENSG00000103381 | up | protein_coding | 3.315724 | 0.001009444 | 0.000779027 |
| PIM1 | ENSG00000137193 | up | protein_coding | 3.315724 | 0.001009444 | 0.000779027 |
| MORF4L1 | ENSG00000185787 | up | protein_coding | 3.315724 | 0.001009444 | 0.000779027 |
| POLR2B | ENSG00000047315 | up | protein_coding | 3.315724 | 0.001009444 | 0.000779027 |
| ARAP2 | ENSG00000047365 | up | protein_coding | 3.315724 | 0.001009444 | 0.000779027 |
| DNAAF5 | ENSG00000164818 | up | protein_coding | 3.315724 | 0.001009444 | 0.000779027 |
| IL1B | ENSG00000125538 | up | protein_coding | 3.315724 | 0.001009444 | 0.000779027 |
| ANXA1 | ENSG00000135046 | up | protein_coding | 3.315724 | 0.001009444 | 0.000779027 |
| NELFE | ENSG00000204356 | up | protein_coding | 3.315724 | 0.001009444 | 0.000779027 |
| EHMT2 | ENSG00000204371 | up | protein_coding | 3.315724 | 0.001009444 | 0.000779027 |
| MYO10 | ENSG00000145555 | up | protein_coding | 1.763182 | 0.001059188 | 0.000824324 |
| NDUFB9 | ENSG00000147684 | up | protein_coding | 1.730761 | 0.00140693 | 0.001096182 |
| DISP1 | ENSG00000154309 | up | protein_coding | 3.248609 | 0.001444075 | 0.00113768 |
| IL1R1 | ENSG00000115594 | up | protein_coding | 3.248609 | 0.001444075 | 0.00113768 |
| PLEKHN1 | ENSG00000187583 | up | protein_coding | 3.248609 | 0.001444075 | 0.00113768 |
| CRYBB2P1 | ENSG00000100058 | up | transcribed_unprocessed_pseudogene | 3.248609 | 0.001444075 | 0.00113768 |
| PSMD12 | ENSG00000197170 | up | protein_coding | 3.248609 | 0.001444075 | 0.00113768 |
| FAM208A | ENSG00000163946 | up | protein_coding | 3.248609 | 0.001444075 | 0.00113768 |
| RREB1 | ENSG00000124782 | up | protein_coding | 3.248609 | 0.001444075 | 0.00113768 |
| KIFAP3 | ENSG00000075945 | up | protein_coding | 3.248609 | 0.001444075 | 0.00113768 |
| DHCR7 | ENSG00000172893 | up | protein_coding | 3.248609 | 0.001444075 | 0.00113768 |
| RNA5S9 | ENSG00000201321 | up | rRNA | 3.248609 | 0.001444075 | 0.00113768 |
| PSMD7 | ENSG00000103035 | up | protein_coding | 3.248609 | 0.001444075 | 0.00113768 |
| DMBT1P1 | ENSG00000176584 | up | transcribed_unprocessed_pseudogene | 3.248609 | 0.001444075 | 0.00113768 |
| QKI | ENSG00000112531 | up | protein_coding | 3.248609 | 0.001444075 | 0.00113768 |
| TADA3 | ENSG00000171148 | up | protein_coding | 3.248609 | 0.001444075 | 0.00113768 |
| CFH | ENSG00000000971 | up | protein_coding | 3.248609 | 0.001444075 | 0.00113768 |
| NIPSNAP2 | ENSG00000146729 | up | protein_coding | 3.248609 | 0.001444075 | 0.00113768 |
| CAMTA1 | ENSG00000171735 | up | protein_coding | 3.248609 | 0.001444075 | 0.00113768 |
| PRSS23 | ENSG00000150687 | up | protein_coding | 3.248609 | 0.001444075 | 0.00113768 |
| ZBTB7B | ENSG00000160685 | up | protein_coding | 3.248609 | 0.001444075 | 0.00113768 |
| MT-CYB | ENSG00000198727 | up | protein_coding | 1.528717 | 0.001637269 | 0.00130412 |
| KMT5A | ENSG00000183955 | up | protein_coding | 2.556732 | 0.001649328 | 0.00131516 |
| DENND1A | ENSG00000119522 | up | protein_coding | 3.17822 | 0.002073015 | 0.001661116 |
| HDGF | ENSG00000143321 | up | protein_coding | 3.17822 | 0.002073015 | 0.001661116 |
| CYR61 | ENSG00000142871 | up | protein_coding | 3.17822 | 0.002073015 | 0.001661116 |
| PARP8 | ENSG00000151883 | up | protein_coding | 3.17822 | 0.002073015 | 0.001661116 |
| HERPUD2 | ENSG00000122557 | up | protein_coding | 3.17822 | 0.002073015 | 0.001661116 |
| TIMP1 | ENSG00000102265 | up | protein_coding | 3.17822 | 0.002073015 | 0.001661116 |
| CELF1 | ENSG00000149187 | up | protein_coding | 3.17822 | 0.002073015 | 0.001661116 |
| SELENOM | ENSG00000198832 | up | protein_coding | 3.17822 | 0.002073015 | 0.001661116 |
| PAF1 | ENSG00000006712 | up | protein_coding | 1.367254 | 0.002198878 | 0.001770575 |
| PDCD7 | ENSG00000090470 | up | protein_coding | 2.500148 | 0.002313897 | 0.001865202 |
| APP | ENSG00000142192 | up | protein_coding | 1.943755 | 0.002349486 | 0.001895933 |
| SLC16A4 | ENSG00000168679 | up | protein_coding | 3.104219 | 0.002978995 | 0.002424642 |
| CXCL9 | ENSG00000138755 | up | protein_coding | 3.104219 | 0.002978995 | 0.002424642 |
| TMTC1 | ENSG00000133687 | up | protein_coding | 3.104219 | 0.002978995 | 0.002424642 |
| FBXL3 | ENSG00000005812 | up | protein_coding | 3.104219 | 0.002978995 | 0.002424642 |
| ZNF688 | ENSG00000229809 | up | protein_coding | 3.104219 | 0.002978995 | 0.002424642 |
| DDX3X | ENSG00000215301 | up | protein_coding | 3.104219 | 0.002978995 | 0.002424642 |
| ASAP2 | ENSG00000151693 | up | protein_coding | 3.104219 | 0.002978995 | 0.002424642 |
| ATP6V1G1 | ENSG00000136888 | up | protein_coding | 3.104219 | 0.002978995 | 0.002424642 |
| DNM1L | ENSG00000087470 | up | protein_coding | 3.104219 | 0.002978995 | 0.002424642 |
| CNBP | ENSG00000169714 | up | protein_coding | 3.104219 | 0.002978995 | 0.002424642 |
| MTURN | ENSG00000180354 | up | protein_coding | 3.104219 | 0.002978995 | 0.002424642 |
| ECM2 | ENSG00000106823 | up | protein_coding | 3.104219 | 0.002978995 | 0.002424642 |
| DHX38 | ENSG00000140829 | up | protein_coding | 3.104219 | 0.002978995 | 0.002424642 |
| CTNND1 | ENSG00000198561 | up | protein_coding | 3.104219 | 0.002978995 | 0.002424642 |
| NDUFA12 | ENSG00000184752 | up | protein_coding | 3.104219 | 0.002978995 | 0.002424642 |
| SYNCRIP | ENSG00000135316 | up | protein_coding | 2.441254 | 0.003214861 | 0.002640379 |
| LDHB | ENSG00000111716 | up | protein_coding | 2.441254 | 0.003214861 | 0.002640379 |
| MYL12B | ENSG00000118680 | up | protein_coding | 1.593257 | 0.004061688 | 0.00334118 |
| BRMS1L | ENSG00000100916 | up | protein_coding | 3.026217 | 0.004264425 | 0.003537618 |
| B4GALT5 | ENSG00000158470 | up | protein_coding | 3.026217 | 0.004264425 | 0.003537618 |
| PTGES3 | ENSG00000110958 | up | protein_coding | 3.026217 | 0.004264425 | 0.003537618 |
| CD300E | ENSG00000186407 | up | protein_coding | 3.026217 | 0.004264425 | 0.003537618 |
| AC092378.1 | ENSG00000245768 | up | lincRNA | 3.026217 | 0.004264425 | 0.003537618 |
| DENND2A | ENSG00000146966 | up | protein_coding | 3.026217 | 0.004264425 | 0.003537618 |
| RAB11FIP1 | ENSG00000156675 | up | protein_coding | 3.026217 | 0.004264425 | 0.003537618 |
| TPP1 | ENSG00000166340 | up | protein_coding | 3.026217 | 0.004264425 | 0.003537618 |
| EZR | ENSG00000092820 | up | protein_coding | 3.026217 | 0.004264425 | 0.003537618 |
| BAX | ENSG00000087088 | up | protein_coding | 3.026217 | 0.004264425 | 0.003537618 |
| IMP4 | ENSG00000136718 | up | protein_coding | 3.026217 | 0.004264425 | 0.003537618 |
| PKHD1 | ENSG00000170927 | up | protein_coding | 3.026217 | 0.004264425 | 0.003537618 |
| P4HA2 | ENSG00000072682 | up | protein_coding | 3.026217 | 0.004264425 | 0.003537618 |
| PARD3B | ENSG00000116117 | up | protein_coding | 3.026217 | 0.004264425 | 0.003537618 |
| CLIC6 | ENSG00000159212 | up | protein_coding | 3.026217 | 0.004264425 | 0.003537618 |
| ARF6 | ENSG00000165527 | up | protein_coding | 2.379854 | 0.004459115 | 0.003730146 |
| NPAS2 | ENSG00000170485 | down | protein_coding | -1.68428 | 0.004891867 | 0.004096407 |
| HIST1H1C | ENSG00000187837 | up | protein_coding | 2.943755 | 0.006103248 | 0.005158571 |
| PNN | ENSG00000100941 | up | protein_coding | 2.943755 | 0.006103248 | 0.005158571 |
| C16orf72 | ENSG00000182831 | up | protein_coding | 2.943755 | 0.006103248 | 0.005158571 |
| PPP1CC | ENSG00000186298 | up | protein_coding | 2.943755 | 0.006103248 | 0.005158571 |
| CASK | ENSG00000147044 | up | protein_coding | 2.943755 | 0.006103248 | 0.005158571 |
| TRIR | ENSG00000123144 | up | protein_coding | 2.943755 | 0.006103248 | 0.005158571 |
| PPP2R2A | ENSG00000221914 | up | protein_coding | 2.943755 | 0.006103248 | 0.005158571 |
| SOX2 | ENSG00000181449 | up | protein_coding | 2.943755 | 0.006103248 | 0.005158571 |
| FPGS | ENSG00000136877 | up | protein_coding | 2.943755 | 0.006103248 | 0.005158571 |
| RNA5-8S4 | ENSG00000278233 | up | rRNA | 2.943755 | 0.006103248 | 0.005158571 |
| C7orf49 | ENSG00000122783 | up | protein_coding | 2.943755 | 0.006103248 | 0.005158571 |
| HLA-F | ENSG00000204642 | up | protein_coding | 2.943755 | 0.006103248 | 0.005158571 |
| EXOC3 | ENSG00000180104 | up | protein_coding | 2.943755 | 0.006103248 | 0.005158571 |
| THRA | ENSG00000126351 | up | protein_coding | 2.943755 | 0.006103248 | 0.005158571 |
| RPS2P48 | ENSG00000233380 | up | processed_pseudogene | 2.943755 | 0.006103248 | 0.005158571 |
| SNN | ENSG00000184602 | up | protein_coding | 2.943755 | 0.006103248 | 0.005158571 |
| APOBR | ENSG00000184730 | up | protein_coding | 2.943755 | 0.006103248 | 0.005158571 |
| HMOX2 | ENSG00000103415 | up | protein_coding | 2.315724 | 0.006160702 | 0.005258025 |
| MUM1 | ENSG00000160953 | up | protein_coding | 2.315724 | 0.006160702 | 0.005258025 |
| PHGDH | ENSG00000092621 | up | protein_coding | 1.206789 | 0.008519459 | 0.007282285 |
| ARHGAP32 | ENSG00000134909 | up | protein_coding | 1.480783 | 0.008742313 | 0.007480379 |
| DNM2 | ENSG00000079805 | up | protein_coding | 2.856292 | 0.008705249 | 0.007516793 |
| FOSL2 | ENSG00000075426 | up | protein_coding | 2.856292 | 0.008705249 | 0.007516793 |
| CMPK2 | ENSG00000134326 | up | protein_coding | 2.856292 | 0.008705249 | 0.007516793 |
| RGL1 | ENSG00000143344 | up | protein_coding | 2.856292 | 0.008705249 | 0.007516793 |
| SPCS2 | ENSG00000118363 | up | protein_coding | 2.856292 | 0.008705249 | 0.007516793 |
| RAB30-AS1 | ENSG00000246067 | up | lincRNA | 2.856292 | 0.008705249 | 0.007516793 |
| HIPK2 | ENSG00000064393 | up | protein_coding | 2.856292 | 0.008705249 | 0.007516793 |
| DDX60L | ENSG00000181381 | up | protein_coding | 2.856292 | 0.008705249 | 0.007516793 |
| MSL3 | ENSG00000005302 | up | protein_coding | 2.856292 | 0.008705249 | 0.007516793 |
| FLI1 | ENSG00000151702 | up | protein_coding | 2.856292 | 0.008705249 | 0.007516793 |
| IMPG1 | ENSG00000112706 | up | protein_coding | 2.856292 | 0.008705249 | 0.007516793 |
| AKAP11 | ENSG00000023516 | up | protein_coding | 2.856292 | 0.008705249 | 0.007516793 |
| SIRT1 | ENSG00000096717 | up | protein_coding | 2.856292 | 0.008705249 | 0.007516793 |
| WDR45B | ENSG00000141580 | up | protein_coding | 2.856292 | 0.008705249 | 0.007516793 |
| CENPB | ENSG00000125817 | up | protein_coding | 2.856292 | 0.008705249 | 0.007516793 |
| ALDH1A3 | ENSG00000184254 | up | protein_coding | 2.856292 | 0.008705249 | 0.007516793 |
| TES | ENSG00000135269 | up | protein_coding | 2.856292 | 0.008705249 | 0.007516793 |
| GIGYF2 | ENSG00000204120 | up | protein_coding | 1.315724 | 0.008787843 | 0.007656886 |
| EGR3 | ENSG00000179388 | up | protein_coding | 2.17822 | 0.01188914 | 0.010369398 |
| CCNB1 | ENSG00000134057 | up | protein_coding | 2.763182 | 0.012453732 | 0.01094304 |
| ADD3 | ENSG00000148700 | up | protein_coding | 2.763182 | 0.012453732 | 0.01094304 |
| KIAA2026 | ENSG00000183354 | up | protein_coding | 2.763182 | 0.012453732 | 0.01094304 |
| GHDC | ENSG00000167925 | up | protein_coding | 2.763182 | 0.012453732 | 0.01094304 |
| MTRNR2L12 | ENSG00000269028 | up | protein_coding | 2.763182 | 0.012453732 | 0.01094304 |
| SEM1 | ENSG00000127922 | up | protein_coding | 2.763182 | 0.012453732 | 0.01094304 |
| CYB5B | ENSG00000103018 | up | protein_coding | 2.763182 | 0.012453732 | 0.01094304 |
| HLA-B | ENSG00000234745 | up | protein_coding | 2.763182 | 0.012453732 | 0.01094304 |
| UBL5 | ENSG00000198258 | up | protein_coding | 2.763182 | 0.012453732 | 0.01094304 |
| NUDT16 | ENSG00000198585 | up | protein_coding | 2.763182 | 0.012453732 | 0.01094304 |
| RALY | ENSG00000125970 | up | protein_coding | 2.763182 | 0.012453732 | 0.01094304 |
| CCDC12 | ENSG00000160799 | up | protein_coding | 2.763182 | 0.012453732 | 0.01094304 |
| AL031963.1 | ENSG00000228170 | up | antisense_RNA | 2.763182 | 0.012453732 | 0.01094304 |
| TMED2 | ENSG00000086598 | up | protein_coding | 2.763182 | 0.012453732 | 0.01094304 |
| SET | ENSG00000119335 | up | protein_coding | 2.104219 | 0.016348215 | 0.014500156 |
| SARS | ENSG00000031698 | up | protein_coding | 2.104219 | 0.016348215 | 0.014500156 |
| PSME4 | ENSG00000068878 | up | protein_coding | 2.104219 | 0.016348215 | 0.014500156 |
| IL6ST | ENSG00000134352 | up | protein_coding | 2.663647 | 0.017740951 | 0.015912861 |
| TUBA1C | ENSG00000167553 | up | protein_coding | 2.663647 | 0.017740951 | 0.015912861 |
| NUCKS1 | ENSG00000069275 | up | protein_coding | 2.663647 | 0.017740951 | 0.015912861 |
| CEP170 | ENSG00000143702 | up | protein_coding | 2.663647 | 0.017740951 | 0.015912861 |
| VPS54 | ENSG00000143952 | up | protein_coding | 2.663647 | 0.017740951 | 0.015912861 |
| CPS1 | ENSG00000021826 | up | protein_coding | 2.663647 | 0.017740951 | 0.015912861 |
| PTOV1 | ENSG00000104960 | up | protein_coding | 2.663647 | 0.017740951 | 0.015912861 |
| GGPS1 | ENSG00000152904 | up | protein_coding | 2.663647 | 0.017740951 | 0.015912861 |
| USP24 | ENSG00000162402 | up | protein_coding | 2.663647 | 0.017740951 | 0.015912861 |
| VDAC3 | ENSG00000078668 | up | protein_coding | 2.663647 | 0.017740951 | 0.015912861 |
| NPLOC4 | ENSG00000182446 | up | protein_coding | 2.663647 | 0.017740951 | 0.015912861 |
| SATB1 | ENSG00000182568 | up | protein_coding | 2.663647 | 0.017740951 | 0.015912861 |
| SCMH1 | ENSG00000010803 | up | protein_coding | 2.663647 | 0.017740951 | 0.015912861 |
| PDK4 | ENSG00000004799 | up | protein_coding | 2.663647 | 0.017740951 | 0.015912861 |
| RNA5-8SP4 | ENSG00000201966 | up | rRNA | 2.663647 | 0.017740951 | 0.015912861 |
| GRB10 | ENSG00000106070 | up | protein_coding | 2.663647 | 0.017740951 | 0.015912861 |
| UGP2 | ENSG00000169764 | up | protein_coding | 2.663647 | 0.017740951 | 0.015912861 |
| IDO1 | ENSG00000131203 | up | protein_coding | 2.663647 | 0.017740951 | 0.015912861 |
| ATP5F1 | ENSG00000116459 | up | protein_coding | 2.663647 | 0.017740951 | 0.015912861 |
| RBMS2 | ENSG00000076067 | up | protein_coding | 2.663647 | 0.017740951 | 0.015912861 |
| ARHGAP19 | ENSG00000213390 | up | protein_coding | 2.556732 | 0.025307926 | 0.023107237 |
| IGF2R | ENSG00000197081 | up | protein_coding | 2.556732 | 0.025307926 | 0.023107237 |
| PREX1 | ENSG00000124126 | up | protein_coding | 2.556732 | 0.025307926 | 0.023107237 |
| Y_RNA | ENSG00000202224 | up | misc_RNA | 2.556732 | 0.025307926 | 0.023107237 |
| SNTB1 | ENSG00000172164 | up | protein_coding | 2.556732 | 0.025307926 | 0.023107237 |
| C9orf72 | ENSG00000147894 | up | protein_coding | 2.556732 | 0.025307926 | 0.023107237 |
| WTIP | ENSG00000142279 | up | protein_coding | 2.556732 | 0.025307926 | 0.023107237 |
| VTRNA1-1 | ENSG00000199990 | up | misc_RNA | 2.556732 | 0.025307926 | 0.023107237 |
| NT5C3A | ENSG00000122643 | up | protein_coding | 2.556732 | 0.025307926 | 0.023107237 |
| RN7SL471P | ENSG00000263426 | up | misc_RNA | 2.556732 | 0.025307926 | 0.023107237 |
| IRF1 | ENSG00000125347 | up | protein_coding | 2.556732 | 0.025307926 | 0.023107237 |
| AC009779.8 | ENSG00000272937 | up | transcribed_unprocessed_pseudogene | 2.556732 | 0.025307926 | 0.023107237 |
| EIF2S2 | ENSG00000125977 | up | protein_coding | 2.556732 | 0.025307926 | 0.023107237 |
| ANK2 | ENSG00000145362 | up | protein_coding | 2.556732 | 0.025307926 | 0.023107237 |
| LONRF2 | ENSG00000170500 | up | protein_coding | 2.556732 | 0.025307926 | 0.023107237 |
| TCP1 | ENSG00000120438 | up | protein_coding | 1.943755 | 0.03044147 | 0.02807233 |
| PPP1CB | ENSG00000213639 | up | protein_coding | 1.943755 | 0.03044147 | 0.02807233 |
| TRIOBP | ENSG00000100106 | up | protein_coding | 2.441254 | 0.03610211 | 0.03349648 |
| AC022509.2 | ENSG00000256234 | up | antisense_RNA | 2.441254 | 0.03610211 | 0.03349648 |
| MECOM | ENSG00000085276 | up | protein_coding | 2.441254 | 0.03610211 | 0.03349648 |
| CRKL | ENSG00000099942 | up | protein_coding | 2.441254 | 0.03610211 | 0.03349648 |
| SSH1 | ENSG00000084112 | up | protein_coding | 2.441254 | 0.03610211 | 0.03349648 |
| NORAD | ENSG00000260032 | up | lincRNA | 2.441254 | 0.03610211 | 0.03349648 |
| AL121983.1 | ENSG00000224977 | up | lincRNA | 2.441254 | 0.03610211 | 0.03349648 |
| AC078846.1 | ENSG00000273329 | up | lincRNA | 2.441254 | 0.03610211 | 0.03349648 |
| AC106872.5 | ENSG00000248632 | up | processed_pseudogene | 2.441254 | 0.03610211 | 0.03349648 |
| PIM3 | ENSG00000198355 | up | protein_coding | 2.441254 | 0.03610211 | 0.03349648 |
| FAM131B | ENSG00000159784 | up | protein_coding | 2.441254 | 0.03610211 | 0.03349648 |
| SRRM2 | ENSG00000167978 | up | protein_coding | 1.379854 | 0.048801975 | 0.045534364 |
